# Supplementary material for: Interplay of EGFR, JNK, and ROS signaling in soma-germline communication in the Drosophila testis
Source: Stem Cell Reports. 2025 Oct 16;20(11):102676. doi: 10.1016/j.stemcr.2025.102676 (PMC12790739; doi:10.1016/j.stemcr.2025.102676)
Supplement: Document S1. Figures S1–S7 and supplemental methods [file mmc1.pdf]

Stem Cell Reports, Volume *20*

Supplemental Information

Interplay of EGFR, JNK, and ROS signaling in soma-germline communication in the *Drosophila* testis

Maria Alvarez and Fani Papagiannouli

SUPPLEMENTAL METHODS

Fly stocks and husbandry

The following stocks were from the Bloomington Stock Center (BL) Indiana: *UAS-scrib-RNAi*$^{TRiP.HMS01490}$, *UAS-AP2α (adaptin)-RNAi*$^{TRiP.HMS00653}$, *UAS-shi-RNAi*$^{TRiP.JF03133}$, *UAS-EGFR*^{CA} (BL9533), *UAS-mCD8-GFP* (BL5139), *Pin/CyO; UAS-mCD8-GFP* (BL5130), *αtub84B-GAL80*^{ts}*/TM2* (BL7017), *αtub-GAL80*^{ts}*; TM2/TM6B,Tb* (BL7019), *UAS-Basket-RNAi*$^{TRiP.JF01275}$ (BL31323), *UAS-Rab35-RNAi*$^{TRiP.JF02978}$ (BL28342), *UAS-Ask1-RNAi*$^{TRiP.HMS300464}$ (BL32464), *UAS-wengen-RNAi*$^{TRiP.HMC03962}$ (BL55275), *UAS-Nox-RNAi*$^{TRiP.HMS00691}$ (BL32902), *UAS-Duox-RNAi*$^{TRiP.HMS00629}$, (BL32846). The following stocks used in this study were from the Vienna *Drosophila* RNAi Center (VDRC) Austria: *UAS-dlg-RNAi*^{v41134}*, UAS-dlg-RNAi*^{v41136}*/TM3, UAS-lgl-RNAi*^{v109604}*, UAS-lgl-RNAi*^{v51247}*, UAS-Chc-RNAi*^{v103383}*, UAS-Chc-RNAi*^{v32666}*, UAS-p38a-RNAi*^{v342386}*, UAS-grindewald-RNAi*^{v104538}*, UAS-lic-RNAi*^{v106822}*, Rab35-FlyFos (v318284), wgn-FlyFos (v318644)*. All RNAi stocks used in this study have been effective in knocking down the corresponding genes in previous studies (Brantley and Fuller, 2019; Fujisawa et al., 2020; Papagiannouli et al., 2019; Patel et al., 2019).

The *c587-GAL4* was obtained from Margaret Fuller, JNK reporter *TRE-GFP (attP16)* thereafter mentioned as *puc::TRE-GFP* (Chatterjee and Bohmann, 2012) was obtained from David Bilder. Other fly stocks used in this study are described in FlyBase (www.flybase.org). Genomics EGFR-GFP transgenic fly lines were a gift of Bassem Hassan (Zschatzsch et al., 2014). All *UAS-gene*^{RNAi} stocks are referred to in the text as *gene*^{RNAi} for simplicity reasons. Knockdowns were performed using the *UAS-GAL4* system (Brand and Perrimon, 1993) by combining the *UAS-RNAi* fly lines with the cell-type specific *c587-GAL4* driver (Kai and Spradling, 2004) and *αtub-Gal80*^{ts} (Lee and Luo, 1999).

For the phenotypic analysis in adult *Drosophila* testes: *c587*-GAL4; *αtub-Gal80*^{ts}*; UAS-mCD8-GFP* or *c587*-GAL4*; αtub-Gal80*^{ts} flies were crossed to *UAS-gene*^{RNAi} flies. Crosses were raised at 18°C until adult flies hatched. Then adult males 1-3 days old with the correct genotype (along with few females in order to mate) were shifted at 30°C for 4 or 7 days depending on the experimental needs and the phenotypes were analysed. To control for possible effects of multiple *UAS* constructs limiting the effectiveness of the *GAL4* driver, control flies carried the same number of *UAS* transgenes using a *UAS-mCD8-GFP* (we could not use a *UAS-GFP-RNAi* line, as this would interfere with the *puc::TRE-GFP*).

Immunofluorescence staining and microscopy

The monoclonal antibodies used in this study: anti-Vasa (1/10; rat), anti-Mmp1 antibodies 3B8D12, 5H7B11, 3A6B4 (each used in 1/10 dilution) were obtained from the Developmental Studies Hybridoma Bank developed under the auspices of the NICHD and maintained by The University of

Iowa, Department of Biological Sciences, Iowa City, IA 52242. Polyclonal chicken anti-GFP (13970; 1/10,000) was from Abcam, polyclonal rabbit Phospho-p38 MAPK (Thr180/Tyr182) Antibody (9211; 1/200) from Cell Signaling. Filamentous (F-actin) was stained with Alexa Fluor phalloidin 546 (1/300, Thermo Fischer Scientific) and DNA with DAPI (Thermo Fischer Scientific) or in DAPI containing mounting medium. Following secondary antibodies were used: donkey anti-mouse Alexa Fluor-546 and Alexa Fluor-647, donkey anti-rat Alexa Fluor-647, donkey anti-rabbit Alexa Fluor-488 and donkey anti-chicken Alexa Fluor-488 from Thermo Fischer Scientific (1/500).

For whole mount testes immunostaining, testes were dissected in PBS, fixed for 20min in 8% formaldehyde (FA) and rinsed twice in 1% PBX (1% Triton-100x in PBS). Testes were blocked in 5% Bovine Serum Albumin in 1% PBX for 1h. Testes were incubated with primary antibodies over-night at 4°C and the following day with the secondary antibodies for 2h at room temperature (RT) in the dark (Papagiannouli *et al.*, 2019). For testes immunostaining in the presence of GFP, 1% PBT (1% Tween-20 in PBS) was used instead of 1% PBX in all steps. For the TUNEL assay, tissue was processed as described above except that after fixation, the protocol from In Situ Cell Death Detection Kit (TMR Red, Sigma/Roche) was followed (Papagiannouli *et al.*, 2019). For the Mmp1 staining, DHSB antibodies 3B8D12, 5H7B11, 3A6B4 where mixed in equal amounts (1:1:1 ratio) and the mix was used in a 3/10 dilution. Testes were blocked O/N and incubated in primary antibody mix for 72h. Staining with the polyclonal rabbit Phospho-p38 MAPK antibody gave unspecific sticky staining in spermatocytes, in which case stainings were optimised by preabsorbing the antibody for 2 days with *c587>UAS-mCD8-GFP* testes, before use. Stainings with anti-GFP antibody against Wgn-GFP and EGFR-GFP transgenes were extremely weak, thus, to combat this we incubated the testes for 2 days in the blocking solution to prevent unspecific sticky staining in spermatocytes, and we raised the concentration of anti-GFP to 1/600 dilution. Previous studies on nerve axon branches, also observed too low expression levels of the genomic EGFR-GFP transgene to allow analysis at sub-cellular (Zschatzsch *et al.*, 2014). Testes were mounted in ProLong® Gold Antifade with DAPI (P36931; Thermo Fischer Scientific).

Treatment of flies with antioxidant Vitamin C was performed by feeding male flies with food containing 100mM Vitamin C (L-ascorbic acid; Merck – A4403) for the two days before dissection.

Confocal images were obtained using a Zeiss LSM880 with Airy scan (1024x1024px, 225μm image frame) (University of Greenwich at Medway Campus). Pictures were finally processed with Adobe Photoshop 7.0.

Quantification of fluorescent images and Statistical analysis

Quantifications were done using FiJi/ImageJ by measuring "Corrected Total Cell Fluorescence" (CTCF) [CTCF = Integrated Density - (Area of elected cell X Mean fluorescence of background

readings)] using Excel. Statistical analysis was performed in GraphPad/Prism. The control (in most cases *c587-GAL4* or *c587>UAS-mCD8* testes reflecting physiological levels) was compared to each individual sample using the non-normal, non-parametric two-sampled Mann–Whitney test (also known as Wilcoxon test). Comparisons with a P-value ≥0.05 were marked as 'ns' (not significant); *p<0.05; **p<0.01; ***p<0.001; ****p<0.0001.

ROS staining protocol

A Dihydroethidium (DHE) (D11347, 10x1mg, Invitrogen™) 30mM stock solution was prepared using anhydrous DMSO (Sigma-Aldrich, cat. no. 276855) and kept in small aliquots at -20°C until used. Testes were dissected in DMEM or Schneiders medium (both worked equally well in our hands). 30mM stock was diluted to a 30μM (1/1000) working concentration with DMEM medium and mixed well with vortex, giving rise to a pink solution. Testes were incubated in this mix for 7min in the dark, on an orbital shaker at RT. DHE solution was removed and traces were removed by incubating testes 3 times on DMEM medium for 5 min. Testes were fixed for 4min in 8% formaldehyde, rinsed once with PBS, mounted in ProLong® Gold Antifade with DAPI and imaged immediately with confocal microscope.

REFERENCES

Brand, A.H., and Perrimon, N. (1993). Targeted gene expression as a means of altering cell fates and generating dominant phenotypes. Development *118*, 401-415.

Brantley, S.E., and Fuller, M.T. (2019). Somatic support cells regulate germ cell survival through the Baz/aPKC/Par6 complex. Development *146*. 10.1242/dev.169342.

Chatterjee, N., and Bohmann, D. (2012). A versatile PhiC31 based reporter system for measuring AP-1 and Nrf2 signaling in Drosophila and in tissue culture. PLoS One *7*, e34063. 10.1371/journal.pone.0034063.

Fujisawa, Y., Shinoda, N., Chihara, T., and Miura, M. (2020). ROS Regulate Caspase-Dependent Cell Delamination without Apoptosis in the Drosophila Pupal Notum. iScience *23*, 101413. 10.1016/j.isci.2020.101413.

Kai, T., and Spradling, A. (2004). Differentiating germ cells can revert into functional stem cells in Drosophila melanogaster ovaries. Nature *428*, 564-569. 10.1038/nature02436.

Lee, T., and Luo, L. (1999). Mosaic analysis with a repressible cell marker for studies of gene function in neuronal morphogenesis. Neuron *22*, 451-461.

Papagiannouli, F., Berry, C.W., and Fuller, M.T. (2019). The Dlg Module and Clathrin-Mediated Endocytosis Regulate EGFR Signaling and Cyst Cell-Germline Coordination in the Drosophila Testis. Stem cell reports *12*, 1024-1040. 10.1016/j.stemcr.2019.03.008.

Patel, P.H., Penalva, C., Kardorff, M., Roca, M., Pavlovic, B., Thiel, A., Teleman, A.A., and Edgar, B.A. (2019). Damage sensing by a Nox-Ask1-MKK3-p38 signaling pathway mediates regeneration in the adult Drosophila midgut. Nature communications *10*, 4365. 10.1038/s41467-019-12336-w.

Zschatzsch, M., Oliva, C., Langen, M., De Geest, N., Ozel, M.N., Williamson, W.R., Lemon, W.C., Soldano, A., Munck, S., Hiesinger, P.R., et al. (2014). Regulation of branching dynamics by axon-intrinsic asymmetries in Tyrosine Kinase Receptor signaling. eLife *3*, e01699. 10.7554/eLife.01699.

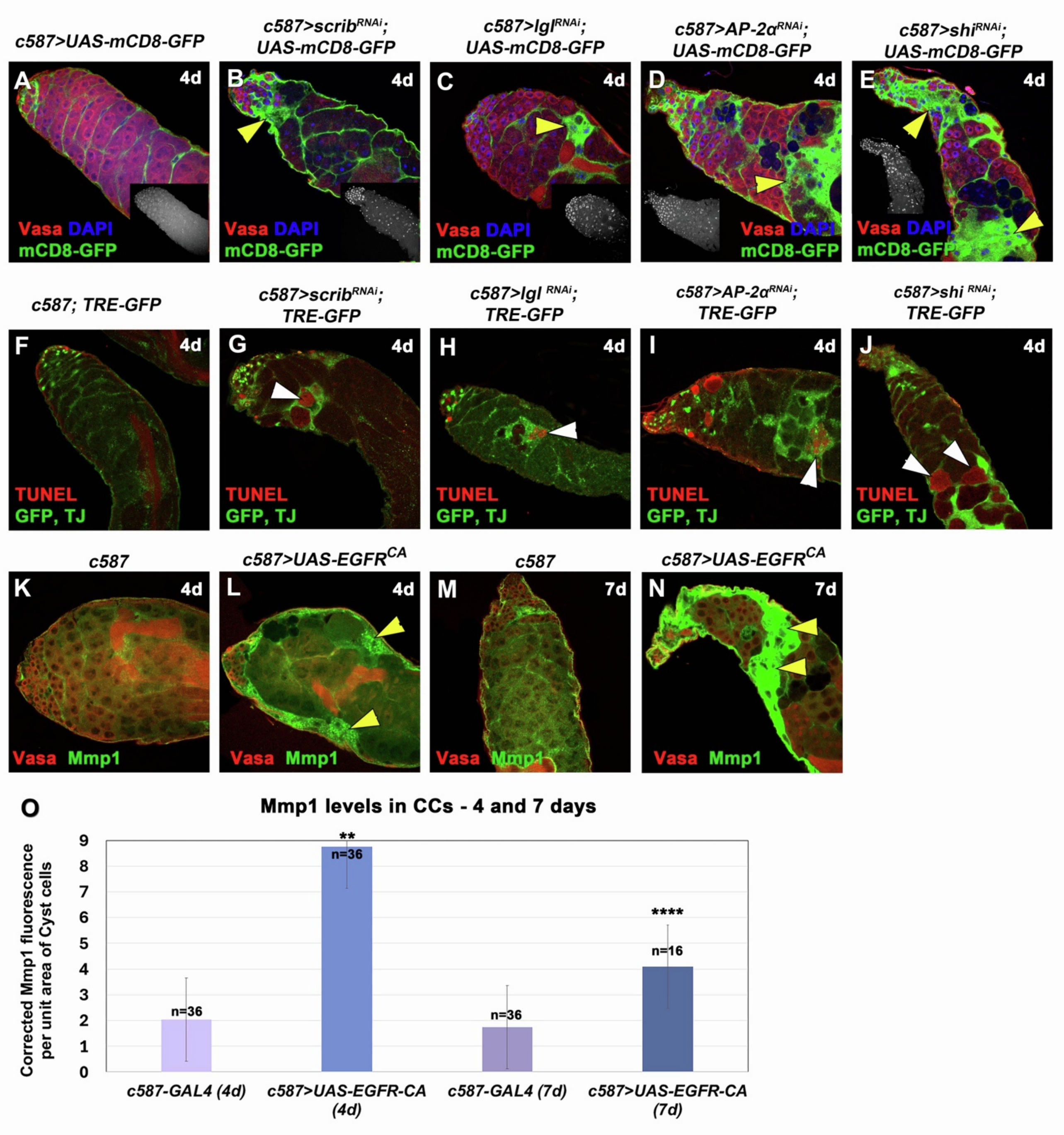

Figure S1: Overactivation of EGFR, also via knockdown of *scrib, lgl, AP-2α* or *shi* function, in cyst cells leads to activation of JNK signaling in cyst cells and apoptosis in the neighbouring germline, related to Figure 1**.** Adult testes of the indicated genotypes in the *Gal80^{ts}* background: **(A-E)** *mCD8-GFP* (green; CCs), Vasa (red; germline), DAPI (blue; nuclei). Yellow arrowheads: mCD8+ CC regions. **(F-J)** TUNEL (red; apoptotic double-strand breaks), AP-1 responsive TRE elements corresponding to JNK reporter *puc* expression levels (*TRE-GFP*) and TJ (early CCs) (green). White arrowheads: dying germ cells (spermatogonia and spermatocytes) surrounded by CCs with upregulated JNK levels. **(K-N)** levels of the Mmp1 protein in CCs (green); Vasa (red; germline). Yellow arrowheads: Mmp1 in CCs. *UAS* activated at 30°C for 4 or 7 days (d). **(O)** Quantification of corrected fluorescent Mmp1 levels in CCs (4- and 7-days activation). Each individual sample was

compared to control (error bars: standard error; ns: not significant; *p<0.05; **p<0.01; ***p<0.001; ****p<0.0001). Numbers (n) in each column represent sample size. Testes oriented with anterior at left. Image frames (A-N): 225μm

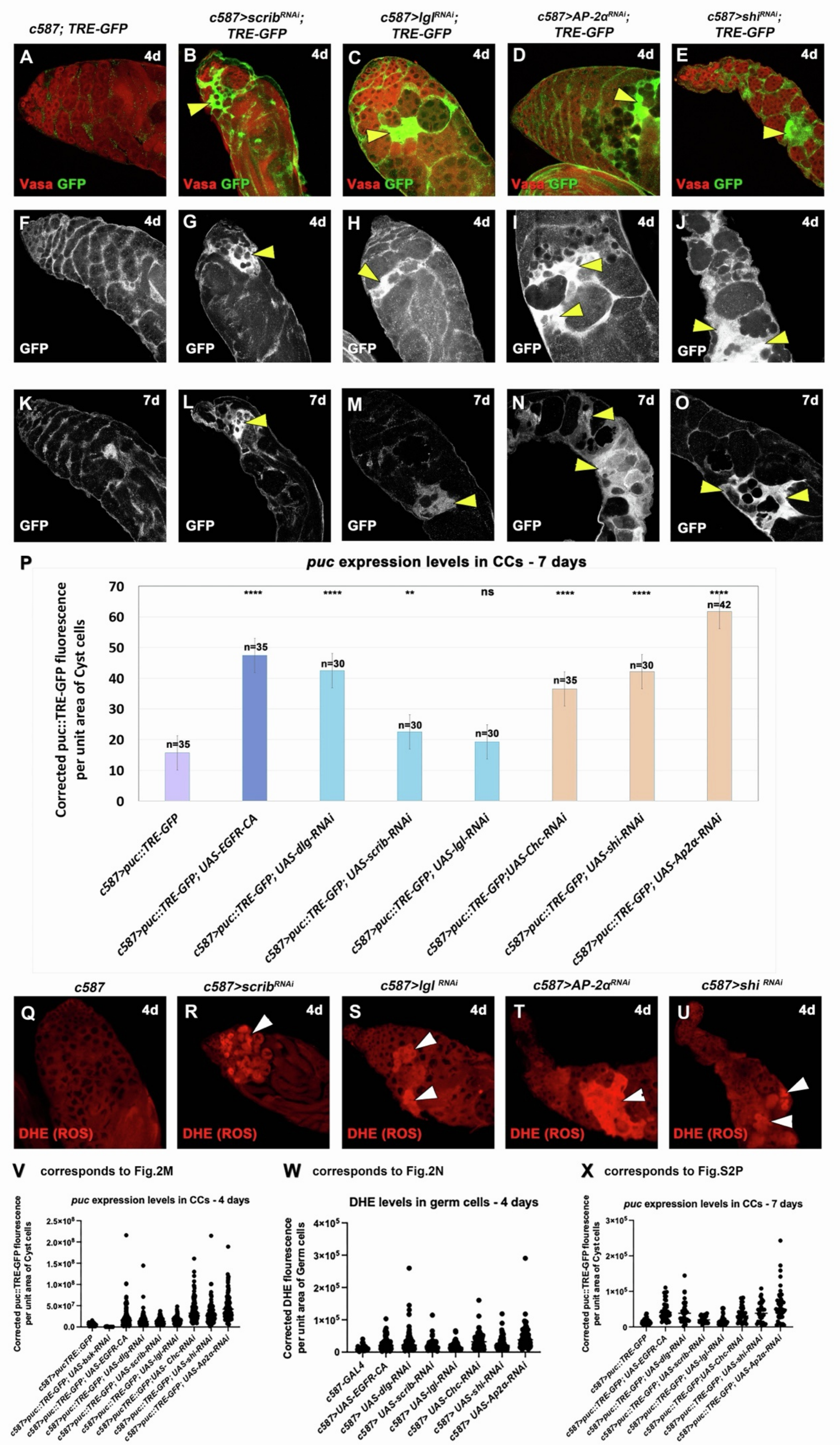

c587; TRE-GFP
c587>scrib^RNAi; TRE-GFP
c587>lgl^RNAi; TRE-GFP
c587>AP-2α^RNAi; TRE-GFP
c587>shi^RNAi; TRE-GFP
Vasa GFP
GFP
4d
7d
P puc expression levels in CCs - 7 days
Corrected puc::TRE-GFP fluorescence per unit area of Cyst cells
c587>puc::TRE-GFP
c587>puc::TRE-GFP; UAS-EGFR-CA
c587>puc::TRE-GFP; UAS-dlg-RNAi
c587>puc::TRE-GFP; UAS-scrib-RNAi
c587>puc::TRE-GFP; UAS-lgl-RNAi
c587>puc::TRE-GFP;UAS-Chc-RNAi
c587>puc::TRE-GFP; UAS-shi-RNAi
c587>puc::TRE-GFP; UAS-Ap2α-RNAi
c587
c587>scrib^RNAi
c587>lgl^RNAi
c587>AP-2α^RNAi
c587>shi^RNAi
DHE (ROS)
V corresponds to Fig.2M
puc expression levels in CCs - 4 days
W corresponds to Fig.2N
DHE levels in germ cells - 4 days
Corrected DHE flourescence per unit area of Germ cells
X corresponds to Fig.S2P
puc expression levels in CCs - 7 days

Figure S2: Overexpression of EGFR or knockdown of *scrib, lgl* or *AP-2α, shi* function, in cyst cells leads to increased levels of JNK signaling in the cyst cells and ROS in the germline, related to Figure 2**.** Adult testes of the indicated genotypes in the $Gal80^{ts}$ background. **(A-O)** *TRE-GFP* reflects expression levels of JNK reporter *puc* in CCs for 4 days (A-J) and 7 days (K-O) activation. (A-E) *TRE-GFP* (green; CCs); Vasa (red; germline). (F-O) show the *puc::TRE-GFP* levels only (white) in directly comparable raw images. Yellow arrowheads: regions of *puc::TRE* overactivation in CCs. **(P)** Quantification of corrected fluorescent *puc::TRE-GFP* levels in CCs (7 days activation). Each individual sample was compared to control (error bars: standard error; ns: not significant; *p<0.05; **p<0.01; ***p<0.001; ****p<0.0001). Numbers (n) in each column represent sample size. **(Q-U)** DHE (red; germ cells) reflects ROS activation in the germ cells. White arrowheads: representative areas of ROS activation in the germline. *UAS* activated at 30°C for 4 days (4d). **(V-X)** Dotted plots for quantifications of Figures 2M, 2N and the S2P (above). Testes oriented with anterior at left. Image frames (A-O, Q-U): 225μm

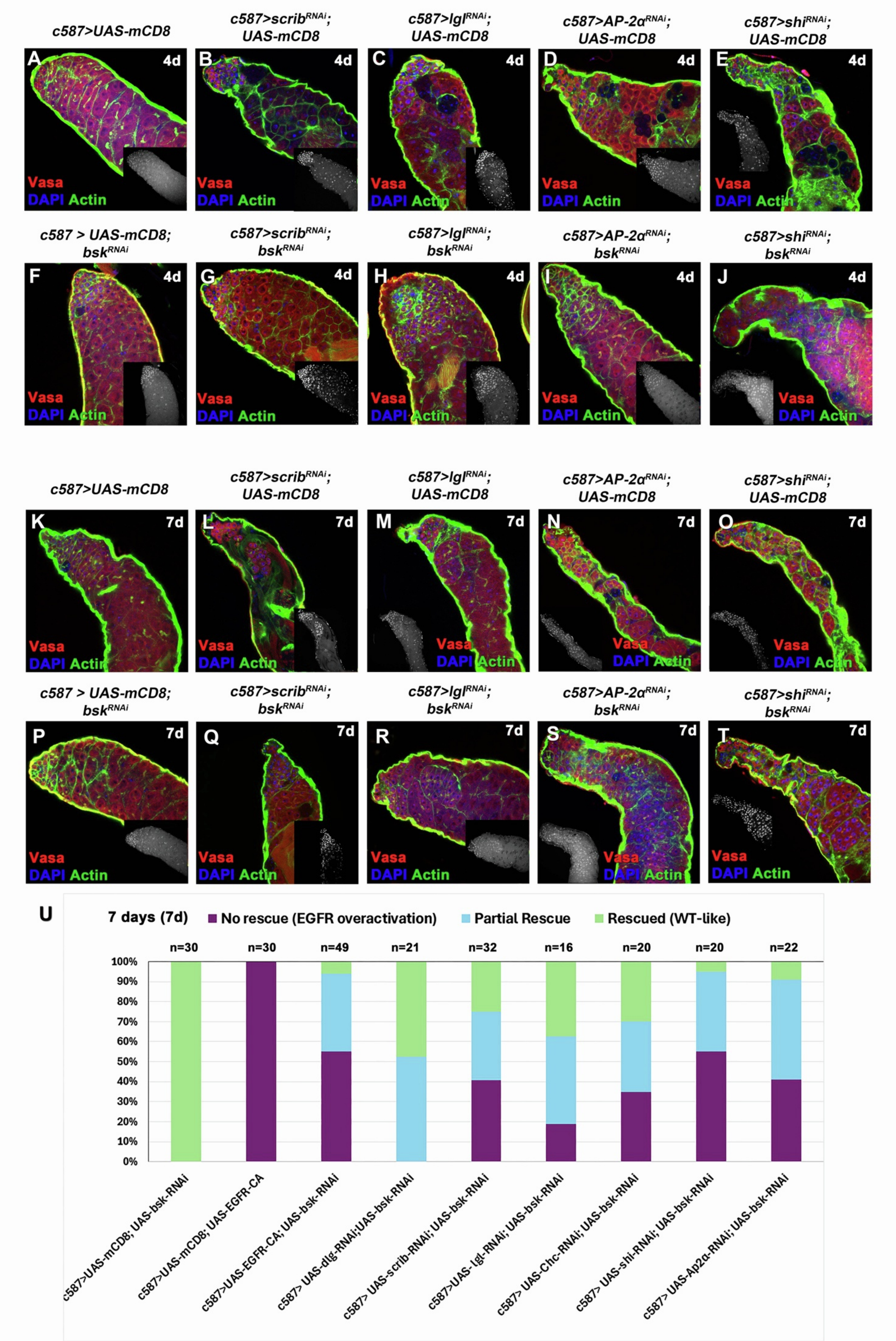
c587>UAS-mCD8
c587>scribRNAi; UAS-mCD8
c587>lglRNAi; UAS-mCD8
c587>AP-2αRNAi; UAS-mCD8
c587>shiRNAi; UAS-mCD8
c587 > UAS-mCD8; bskRNAi
c587>scribRNAi; bskRNAi
c587>lglRNAi; bskRNAi
c587>AP-2αRNAi; bskRNAi
c587>shiRNAi; bskRNAi
Vasa
DAPI Actin
4d
7d
U
7 days (7d)
No rescue (EGFR overactivation)
Partial Rescue
Rescued (WT-like)
n=30
n=30
n=49
n=21
n=32
n=16
n=20
n=20
n=22
100%
90%
80%
70%
60%
50%
40%
30%
20%
10%
0%
c587>UAS-mCD8; UAS-bsk-RNAi
c587>UAS-mCD8; UAS-EGFR-CA
c587>UAS-EGFR-CA; UAS-bsk-RNAi
c587> UAS-dlg-RNAi;UAS-bsk-RNAi
c587> UAS-scrib-RNAi; UAS-bsk-RNAi
c587>UAS- lgl-RNAi; UAS-bsk-RNAi
c587> UAS-Chc-RNAi; UAS-bsk-RNAi
c587> UAS-shi-RNAi; UAS-bsk-RNAi
c587> UAS-Ap2α-RNAi; UAS-bsk-RNAi

Figure S3: Knocking down the JUN kinase *bsk* in cyst cells, can partially rescue the germ cell death phenotype observed upon EGFR overactivation, related to Figure 3**. (A-T)** Adult testes of the indicated genotypes in the *Gal80^ts^* background: Vasa (red; germline), DAPI (blue; nuclei) and Actin stained with phalloidin (here shown in the green channel; hub, CySCs, CCs and germline fusome) also in flies containing the *mCD8-GFP* transgene (since the GFP is not shown here). *UAS* activated at 30°C for 4 (A-J) and 7 (K-T) days (d). **(U)** Quantifications of the different phenotypic classes accompanying each genotype, organized in order of phenotypic strength (4- and 7- days activation). Numbers (n) in each column represent sample size. Testes oriented with anterior at left. Image frames (A-T): 225μm.

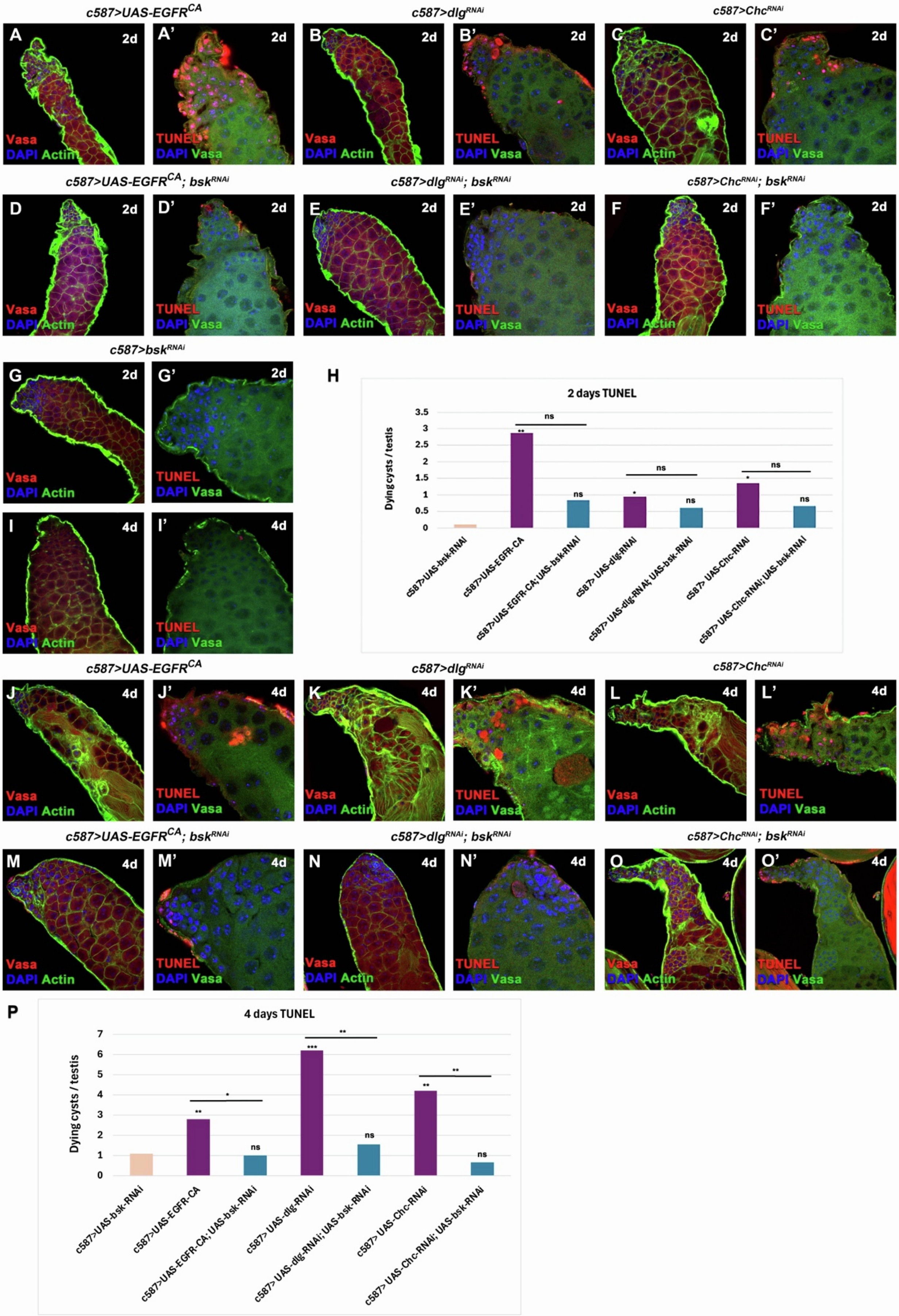
c587>UAS-EGFR^CA
c587>dlg^RNAi
c587>Chc^RNAi
c587>UAS-EGFR^CA; bsk^RNAi
c587>dlg^RNAi; bsk^RNAi
c587>Chc^RNAi; bsk^RNAi
c587>bsk^RNAi
Vasa DAPI Actin
TUNEL DAPI Vasa
2d
4d
2 days TUNEL
Dying cysts / testis
c587>UAS-bsk-RNAi
c587>UAS-EGFR-CA
c587>UAS-EGFR-CA; UAS-bsk-RNAi
c587> UAS-dlg-RNAi
c587> UAS-dlg-RNAi; UAS-bsk-RNAi
c587> UAS-Chc-RNAi
c587> UAS-Chc-RNAi; UAS-bsk-RNAi
ns
4 days TUNEL

Figure S4: Lowering the levels of the JUN kinase *basket* in cyst cells showed significant reduction in germ cell death (GCD), related to Figure 4**.** Adult testes of the indicated genotypes in the *Gal80^ts^* background. *UAS* activation was performed for 2 days in (A-G') and 4 days in (I-O')**. (A-G** and **I-O)** Vasa (red; germline), DAPI (blue; nuclei) and Actin stained with phalloidin (green; hub, CySCs, CCs and germline fusome) (image frames 225μm) show the overall testis structure in EGFR overactivation (A-C, J-L) vs *bsk*-RNAi rescue context (D-F, M-O) and control (G, I). Image frames 225μm. **(A'-G'** and **I'-O')** show higher magnification of (A-G and I-O) at 112.5μm with TUNEL (red; apoptosis), Vasa (here green; germline), DAPI (blue; nuclei) for a better visualization of the dying germ cell cysts. **(H)** and **(P)** Quantifications of dying germline cysts per testes accompanying each genotype after 2- and 4- days of *UAS* activation, respectively. Each genotype was individually compared to control *c587>UAS-bsk-RNAi*, while EGFR overactivation phenotypes (columns 2, 4, 6) were compared to the corresponding bsk-mediated rescues (columns 3, 5, 7) (error bars: standard error; ns: not significant; *p<0.05; **p<0.01). Testes oriented with anterior at left.

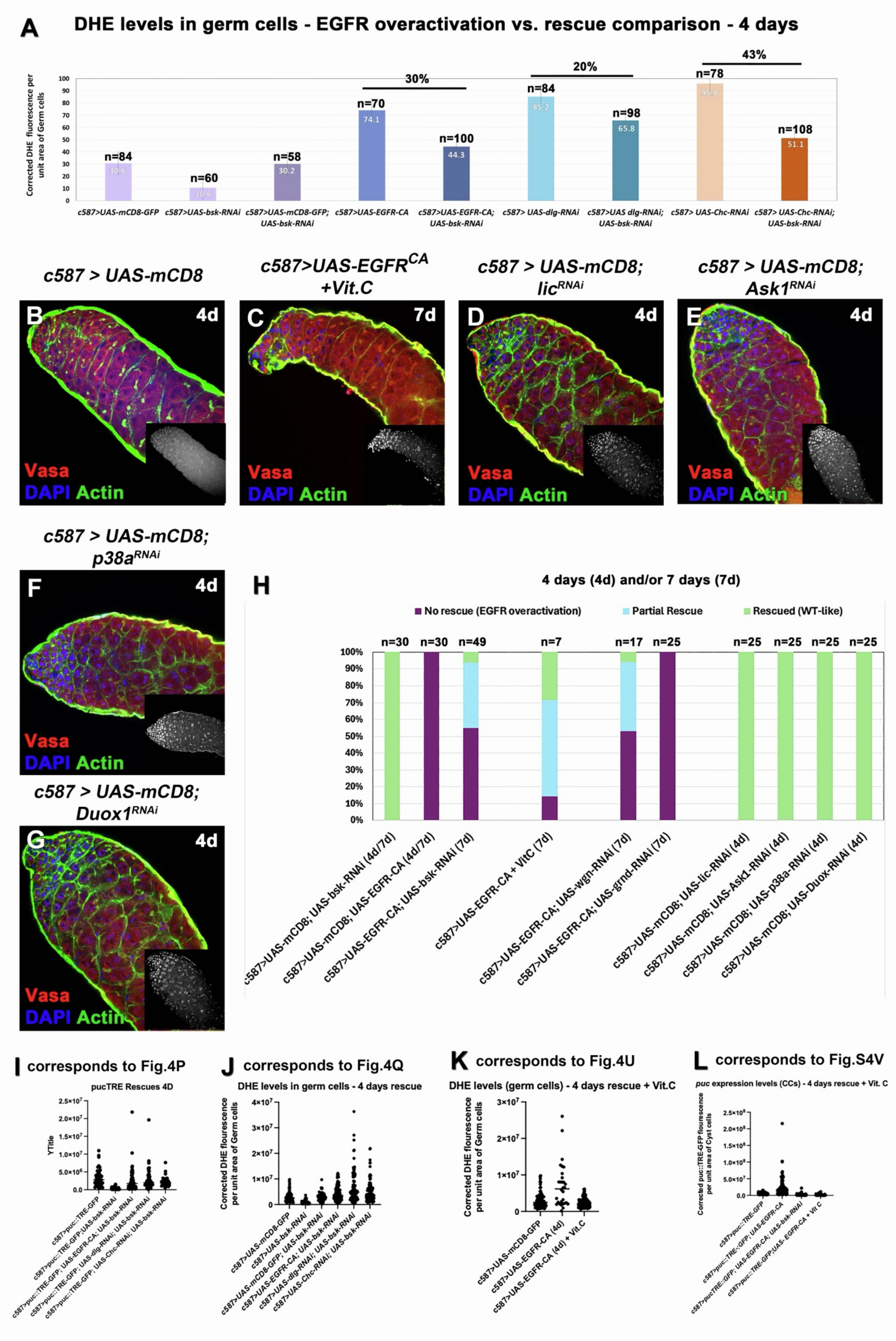
A
DHE levels in germ cells - EGFR overactivation vs. rescue comparison - 4 days
Corrected DHE fluorescence per unit area of Germ cells
30%
20%
43%
n=84
n=60
n=58
n=70
n=100
n=84
n=98
n=78
n=108
c587>UAS-mCD8-GFP
c587>UAS-bsk-RNAi
c587>UAS-mCD8-GFP; UAS-bsk-RNAi
c587>UAS-EGFR-CA
c587>UAS-EGFR-CA; UAS-bsk-RNAi
c587> UAS-dlg-RNAi
c587>UAS dlg-RNAi; UAS-bsk-RNAi
c587> UAS-Chc-RNAi
c587> UAS-Chc-RNAi; UAS-bsk-RNAi
c587 > UAS-mCD8
c587>UAS-EGFR^CA +Vit.C
c587 > UAS-mCD8; lic^RNAi
c587 > UAS-mCD8; Ask1^RNAi
B 4d
C 7d
D 4d
E 4d
Vasa
DAPI Actin
c587 > UAS-mCD8; p38a^RNAi
F 4d
c587 > UAS-mCD8; Duox1^RNAi
G 4d
H
4 days (4d) and/or 7 days (7d)
No rescue (EGFR overactivation)
Partial Rescue
Rescued (WT-like)
n=30 n=30 n=49 n=7 n=17 n=25 n=25 n=25 n=25 n=25
c587>UAS-mCD8; UAS-bsk-RNAi (4d/7d)
c587>UAS-mCD8; UAS-EGFR-CA (4d/7d)
c587>UAS-EGFR-CA; UAS-bsk-RNAi (7d)
c587>UAS-EGFR-CA + VitC (7d)
c587>UAS-EGFR-CA; UAS-wgn-RNAi (7d)
c587>UAS-EGFR-CA; UAS-grnd-RNAi (7d)
c587>UAS-mCD8; UAS-lic-RNAi (4d)
c587>UAS-mCD8; UAS-Ask1-RNAi (4d)
c587>UAS-mCD8; UAS-p38a-RNAi (4d)
c587>UAS-mCD8; UAS-Duox-RNAi (4d)
I corresponds to Fig.4P
pucTRE Rescues 4D
J corresponds to Fig.4Q
DHE levels in germ cells - 4 days rescue
K corresponds to Fig.4U
DHE levels (germ cells) - 4 days rescue + Vit.C
L corresponds to Fig.S4V
puc expression levels (CCs) - 4 days rescue + Vit. C

Figure S5: (A) Double knockdown of the JUN kinase *bsk* with *scrib, lgl, Ap-2α* or *shi* in cyst cells, lowers ROS levels in the germline, related to Figure 5**.** Combined quantifications of ROS levels from Fig.2N and Fig.4Q, comparing DHE levels in “EGFR overactivation” vs *bsk*-rescue backgrounds. Numbers inside the columns represent DHE levels in “EGFR overactivation” genotypes vs. rescues and % reflects this difference for each pair. Numbers (n) above each column represent sample size. As original data in Fig.2N and Fig.4Q, were obtained with a different laser, they were normalised against the *c587* controls to allow the comparison. **(B-G) Treatment with Vitamin C and knockdown of p38 pathway components in cyst cells.** Adult testes of the indicated genotypes in the $Gal80^{ts}$ background. Vasa (red; germline), DAPI (blue; nuclei) and Actin stained with phalloidin (here shown in the green channel; hub, CySCs, CCs and germline fusome) also in flies containing the *mCD8-GFP* transgene (since the GFP is not shown here). *UAS* activated at 30°C for 4 and 7 days (d). **(H)** Quantifications of the different phenotypic classes accompanying each genotype, organized in order of phenotypic strength (4- or 7- days activation) **(Matches Figure 5J).** Each individual sample was compared to control (error bars: standard error; ns: not significant; *p<0.05; **p<0.01; ***p<0.001; ****p<0.0001). Numbers (n) in each column represent sample size. **(I-L)** Dotted plots for quantifications of Figures 4P, 4Q, 4U and the S4V (above). Testes oriented with anterior at left. Image frames (A-G, I-L): 225μm

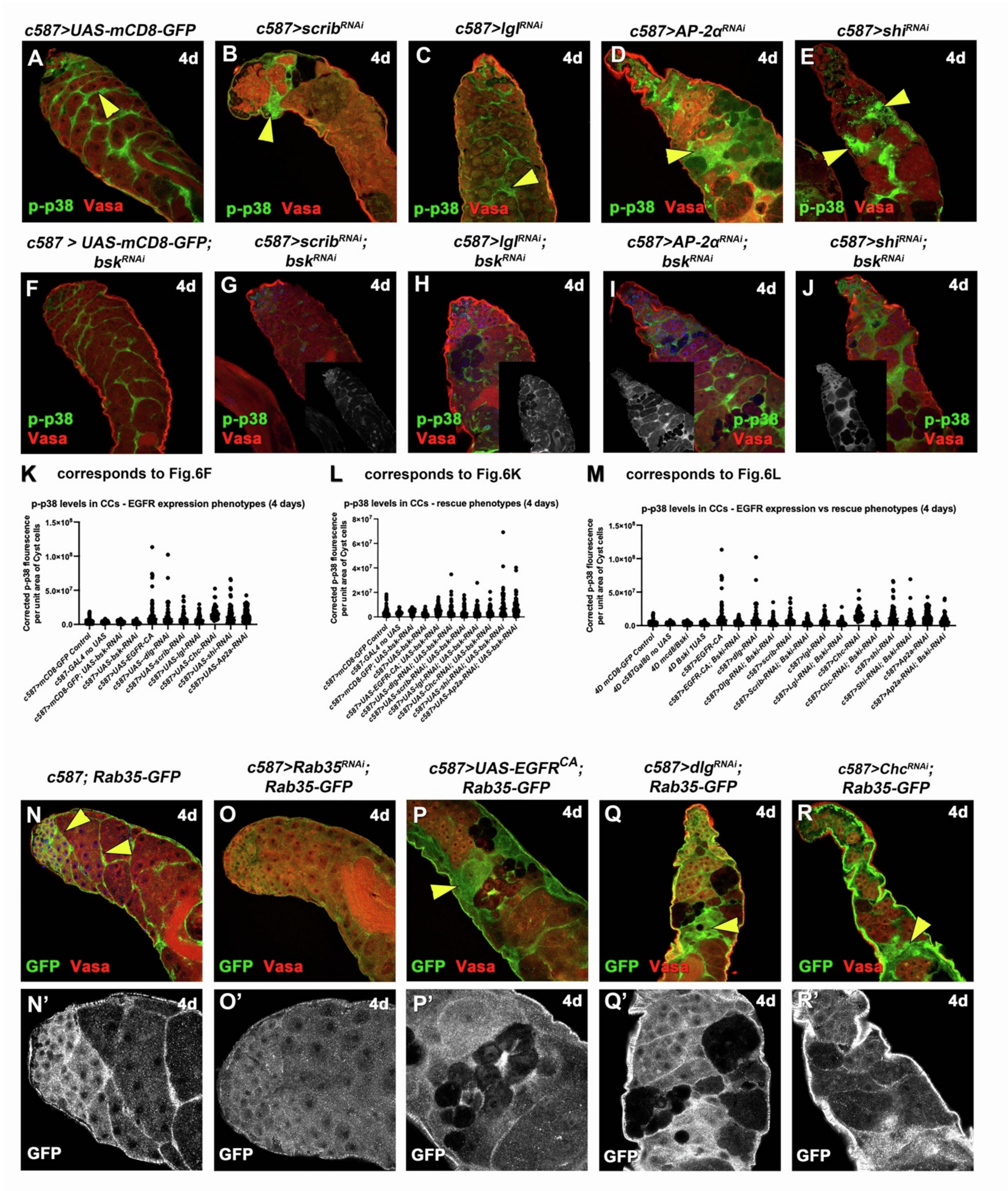

Figure S6: (A-M) Levels of phosphorylated MAPK p38 in EGFR overactivation and rescue phenotypes after knocking down the JUN kinase *basket* in cyst cells, related to Figure 6**.** Adult testes of the indicated genotypes in the *Gal80^ts* background: Vasa (red; germline), DAPI (blue; nuclei) and phosphorylated p38 (p-p38) (green; CCs). Yellow arrowheads point at CCs with high levels of p-p38. Small inset pictures show the p-p38 staining only. **(K-M)** Dotted plots for quantifications of Figures 6F, 6K and 6L.

(N-R') Localization of the Rab35 GTPase in cyst cells and spermatogonia, related to Figure 7**.** Adult testes of the indicated genotypes in the *Gal80*^{ts} background. **(N-R)** Vasa (red; germline), Rab35-GFP (green; CCs and early spermatogonia). Yellow arrowheads point at Rab35 staining in the CCs. **(N'-R')** show the Rab35-GFP staining only. **(O, O')** shows loss of Rab35 staining after knockdown of Rab35 in CCs. *UAS* activated at 30°C for 4 days (4d). Testes oriented with anterior at left. Image frames (A-R) 225μm and (N'-R') 112.5μm

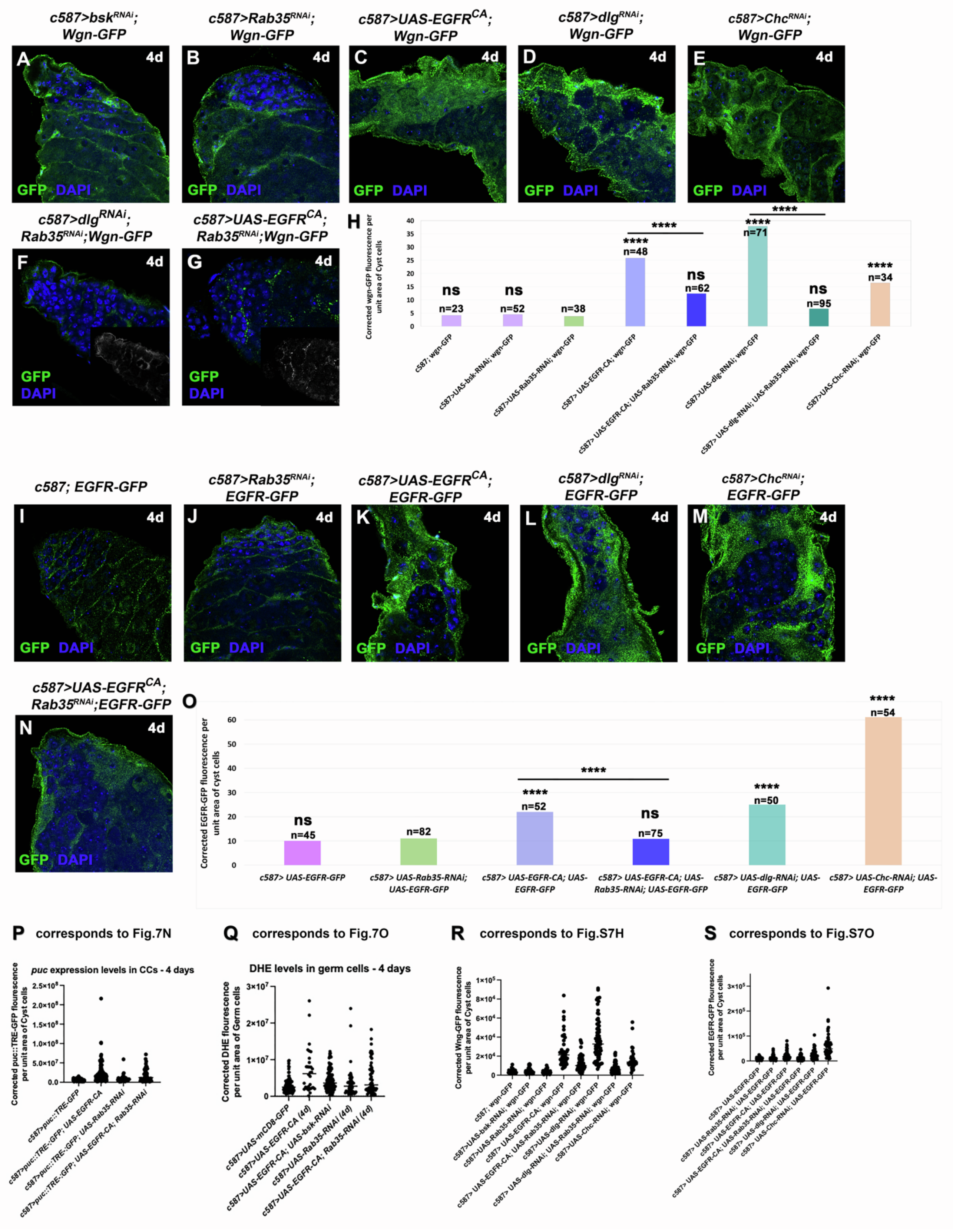

Figure S7: Rab35 is required in cyst cells to regulate Wgn and EGF receptor localization following EGFR overactivation, related to Figure 7**.** Adult testes of the indicated genotypes in the *Gal80*^{ts} background. **(A-G)** Wgn-GFP reflects JNK receptor Wengen localization in CCs. Wgn-GFP (green; CCs) and DAPI (blue; nuclei). Small inset picture in F and G show the Wgn-GFP staining

alone as levels are low to be easily seen in the merged pictures with DAPI. **(I-N)** *EGFR-GFP* reflects endogenous EGFR localization in CCs. EGFR-GFP (green; CCs) and DAPI (blue; nuclei). **(H, O)** Quantification of corrected fluorescent Wgn-GFP and EGFR-GFP levels in CCs, respectively. Each genotype was individually compared to control *c587>UAS-Rab35-RNAi,* while EGFR overactivation phenotypes (columns 4, 6 in S7H and column 3 in S7O) were compared to rescued ones (columns 5, 7 in S7H and column 4 in S7O), and showed significant reduction in the localization of both receptors upon rescue with *Rab35-RNAi* (error bars: standard error; ns: not significant; *p<0.05; **p<0.01; ***p<0.001; ****p<0.0001). Numbers (n) in each column represent sample size. **(P-S)** Dotted plots for quantifications of Figures 7N, 7O and the S7H, S7O (above). Testes oriented with anterior at left.
