# Supplementary material for: Interplay of EGFR, JNK, and ROS signaling in soma-germline communication in the Drosophila testis
Source: Stem Cell Reports. 2025 Oct 16;20(11):102676. doi: 10.1016/j.stemcr.2025.102676 (PMC12790739; doi:10.1016/j.stemcr.2025.102676)
Supplement: Document S2. Article plus supplemental information [file mmc2.pdf]

Stem Cell Reports
Article
OPEN ACCESS

Interplay of EGFR, JNK, and ROS signaling in soma-germline communication in the *Drosophila* testis

Maria Alvarez[1] and Fani Papagiannouli[1,2,*]
[1]Medway School of Pharmacy, Universities of Kent and Greenwich, Chatham, UK
[2]Lead contact
*Correspondence: f.papagiannouli-227@kent.ac.uk
https://doi.org/10.1016/j.stemcr.2025.102676

SUMMARY

Cell communication via signaling exchange plays a pivotal role in multicellular development for building up functional tissues and organs. In the *Drosophila* testis, a pair of somatic cyst cells (CCs) encapsulate the germline that differentiates through close-range EGFR signaling activation. The Dlg/Scrib/Lgl polarity complex and clathrin-mediated endocytosis attenuate EGFR signaling in CCs, and loss of their function leads to EGFR overactivation and death of the neighboring germ cells. Here, we show that EGFR overactivation leads to upregulation of JNK and p38 signaling in CCs and ROS levels in germ cells destined to die. Our data uncover a bidirectional-feedback mechanism between JNK signaling and ROS who regulate each other, while reducing the levels of either JNK or ROS restored germ cell survival. This study provides a framework of how polarity and cellular trafficking regulate the output of multiple signaling responses cell-intrinsically and across cells, to coordinate tissue-specific responses and maintain homeostasis.

INTRODUCTION

Cell-to-cell communication and exchange of short-range signals is crucial for cells to communicate with their neighbors, coordinate their function to the local microenvironment, and build functional tissues and organs. Based on the signals they receive, cells adapt their intrinsic features and signaling machinery to achieve a coordinated output. In the *Drosophila* testis, cell communication between the germline (GL) and the somatic lineage is crucial to produce fertile sperm (Fuller and Spradling, 2007; Zoller and Schulz, 2012). At the anterior-most part of the testis resides the male stem cell niche, a cluster of non-dividing tightly packed somatic cells that build the “hub”. Germline stem cells (GSCs) are organized around the hub, and each GSC is surrounded by a pair of the somatic cyst stem cells (CySCs) (Figure 1A). Upon asymmetric cell division, each GSC produces a new GSC attached to the hub and a daughter gonialblast (GB) that becomes displaced from the hub and enters the differentiation program. Along this differentiation process, the GB enters a stage of four transit-amplifying (TA) mitotic divisions with incomplete cytokinesis, giving rise to 2, 4, 8, and 16 interconnected spermatogonial germ cells. At this stage, germ cells undergo a final round of DNA synthesis, enter meiotic prophase, and turn on the spermatocyte transcription program for meiosis and spermatid differentiation. CySCs also divide asymmetrically to renew themselves and produce two distally located daughter somatic cyst cells (CCs) that cease mitotic divisions and encapsulate the GL from initial differentiation to mature sperm production (Fuller and Spradling, 2007; Zoller and Schulz, 2012). CCs support the progressive steps of GL differentiation through increasing levels of the epidermal growth factor receptor (EGFR) signaling activity (Figure 1B) (Hudson et al., 2013; Papagiannouli, 2022; Sarkar et al., 2007; and references therein). Signals from the GL to the CCs via the EGF ligand Spitz activate the EGFR in CCs and the downstream Ras/MAPK signal transduction pathway, which leads to the double phosphorylated (dp) ERK entering the nucleus. This first step of differentiation is required for germ cells to execute the TA mitotic divisions, while higher levels of EGFR activation are required for germ cells to exit TA divisions and initiate the pre-meiotic program (Hudson et al., 2013). In the absence of EGFR-derived signals, germ cells cannot differentiate and overproliferate as stem-cell-like germ cells, while overactivation of EGFR induces germ cell death (GCD) (Papagiannouli, 2022; Papagiannouli et al., 2019; and references therein), highlighting the importance of fine-tuning signaling levels for a coordinated response.

Our previous work has shown that cortical polarity proteins Discs large (Dlg), Scribble (Scrib), Lethal (2) giant larvae (Lgl), and clathrin-mediated endocytosis (CME) components attenuate EGFR signaling in CCs to promote GL survival and differentiation in adult male testes (Papagiannouli, 2022; Papagiannouli et al., 2019). Dlg, Scrib, and Lgl, collectively called the Dlg module, are highly conserved proteins involved in the establishment and maintenance of apical/basal polarity, signaling regulation, and vesicle and membrane trafficking (reviewed in Papagiannouli, 2022). They are required in the somatic lineage of embryonic gonads and larval testes for the differentiation and survival of the developing *Drosophila* testis (Papagiannouli, 2022; Papagiannouli et al., 2019; and references therein), in a function that is non-tumorigenic,

Published by Elsevier Inc. on behalf of International Society for Stem Cell Research.
This is an open access article under the CC BY license (http://creativecommons.org/licenses/by/4.0/).

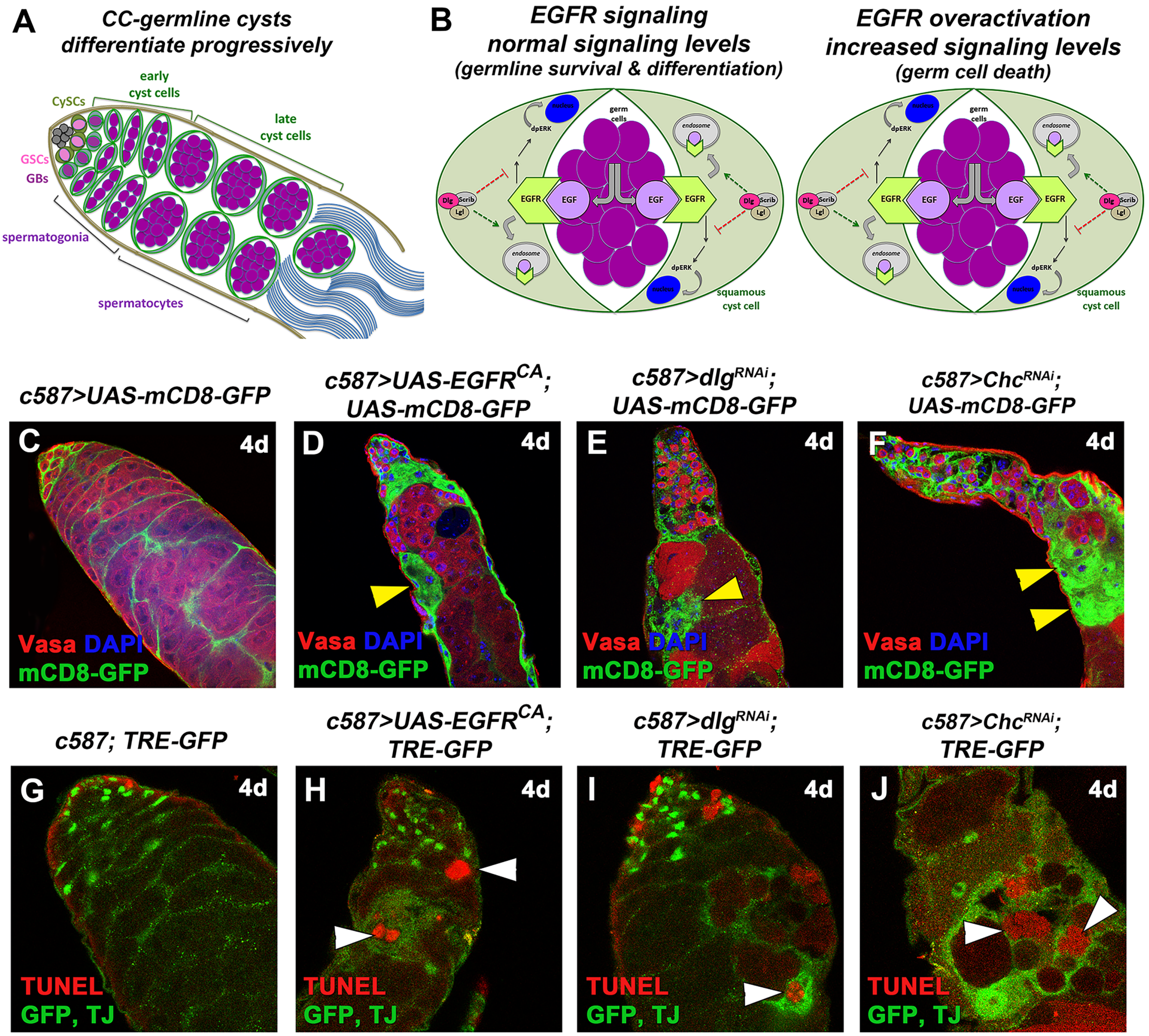

Figure 1. Overactivation of EGFR in CCs leads to activation of JNK signaling in CCs and apoptosis in the neighboring germline (GL)

(A) Diagram of early spermatogenesis in *Drosophila*. GSC, germline stem cell; GB, gonialblast; CySC, somatic cyst stem cell.

(B) Diagram depicting the role of Dlg module and CME components in EGFR signaling regulation. Upon binding the EGF-ligand Spitz from the GL, activated EGFR sends a (unidentified) "GO differentiation" signal that promotes progressive GL differentiation, while Dlg/Scrib/Lgl and CME fine-tune EGFR signaling levels. Loss of Dlg module or CME components leads to EGFR overactivation in CCs that sends now a "Death signal" to the germ cells (black).

(C–F) *mCD8-GFP* (green; CCs), Vasa (red; GL), DAPI (blue; nuclei). Yellow arrowheads: mCD8+ CCs regions.

(G–J) TUNEL (red; apoptotic double-strand breaks), AP-1 responsive TRE elements corresponding to JNK reporter *puc* expression levels (*TRE-GFP*) and TJ (early CC nuclei) (green). White arrowheads: dying germ cells surrounded by CCs with upregulated JNK levels (green). Image frame (C–F): 225 μm and (G–J): 112.5 μm.

unlike epithelia in other tissues (Stephens et al., 2018). On the other hand, clathrin heavy chain (Chc), Shibire (Shi; the *Drosophila* homologue of Dynamin), and AP-2α (also known as α-adaptin) are core components of CME, which have been previously involved in EGFR signaling regulation by removing active EGFR receptor molecules from cell surface membranes in many tissues, while recycling the EGFR back to the membrane via the action of the Rab11-recycling endosome keeps the signaling active (Dobrowolski and De Robertis, 2011; Papagiannouli,

2022; Welz et al., 2014). Knockdown of any of these components in adult testis CCs increases EGFR signaling levels, resulting in cell non-autonomous GCD of both the spermatogonia and spermatocytes they encapsulate. Interestingly, lowering the levels of EGFR signal transduction components can rescue the observed defects and restore germ cell survival (Papagiannouli et al., 2019). Yet, the "death" signal that CCs send to the differentiating germ cells (Figure 1B) as a result of EGFR overactivation is currently unknown.

Reactive oxygen species (ROS) are oxygen derivatives produced from normal aerobic metabolism. In physiological levels, ROS act as secondary signaling molecules in cell biology and redox signaling (Lennicke and Cocheme, 2021; Schieber and Chandel, 2014; Sinenko et al., 2021). Yet, elevated ROS levels can induce oxidative modifications, resulting in cell damage and death termed "oxidative stress". ROS can activate redox-sensitive signals and the mitogen-activated protein kinases (MAPKs) of the Jun N-terminal kinase (JNK) and p38 MAPK signaling pathways, through the MAPKKK apoptotic signal-regulating kinase 1 (Ask1) that is particularly sensitive to ROS oxidative stress (Patel et al., 2019; Sakauchi et al., 2017; Santabarbara-Ruiz et al., 2019; Serras, 2022). JNK and p38 are conserved stress signaling pathways that activate apoptosis as one of their several context-dependent and cell-specific functions (Herrera et al., 2021; Serras, 2022; Wagner and Nebreda, 2009). JNK signaling is initiated through the activation of either of the two tumor necrosis factor (TNF) receptors, Wengen (Wgn) or Grindelwald (Grnd), followed by a series of phosphorylation events leading to phosphorylation of Basket (Bsk), the sole *Drosophila* JUN kinase homologue. In turn, Bsk phosphorylates Kayak and Jun-related antigen (Jrn), the *Drosophila* homologues of Fos and Jun, respectively, which together build the AP-1 transcription factor that activates expression of downstream genes such as *puckered (puc)* and *matrix metalloprotease (Mmp1)* (Uhlirova and Bohmann, 2006). p38 is downstream of a phosphorylation cascade in which Ask1 phosphorylates the serine/threonine kinase licorne (lic), which can activate p38a and p38b but not p38c in *Drosophila*. Phosphorylated p38 can then activate Duox, an NAD(P)H oxidase that promotes ROS activation (Patel et al., 2019; Santabarbara-Ruiz et al., 2019; Takeda et al., 2008).

In this study, we explored the signal that mediates GCD upon EGFR upregulation in *Drosophila* testis CCs. Our data showed that EGFR overactivation in CCs leads to increased levels of JNK and p38 signaling in CCs as well as an increase in ROS oxidative stress levels in the germ cells destined to die. Reducing JNK levels by knocking down the JUN-kinase *bsk* in CCs normalized p38 levels, reduced the levels of ROS in the germ cells, and reversed GCD. Conversely, reducing ROS levels by feeding the flies with the antioxidant vitamin C (Vit.C) restored germ cell survival and JNK signaling in CCs to physiological levels. Our data establish a link between JNK and ROS signaling in the *Drosophila* testis that is coupled to a bidirectional feedback mechanism between the CCs and the differentiating (spermatogonia and spermatocyte) germ cells, also mediated by the JNK receptor Wgn, the p38 MAPK signaling, and the recycling endosome GTPase Rab35.

RESULTS

Overactivation of the EGFR in CCs leads to non-autonomous death of the neighboring differentiating germ cells

In the adult *Drosophila* testis, upon asymmetric GSC division the GB becomes displaced from the hub and enters differentiation consisting of four TA divisions as spermatogonia, followed by a premeiotic stage as spermatocytes, meiosis, and terminal sperm differentiation. Here, the densely packed spermatogonia and spermatocytes were marked with Vasa, while the postmitotic CCs encapsulating them were marked with a membrane(m)CD8-GFP (*UAS-mCD8-GFP*) under the control of the CC lineage *c587-GAL4* driver (Figures 1C and S1A). The nuclei of early CCs (encapsulating primarily spermatogonia) were stained for the Traffic-Jam (TJ) transcription factor (Figures 1G and S1F). The function of EGFR, Dlg-module, and CME components was impaired in CCs using the *c587-GAL4* driver together with *UAS* transgenes to overexpress a constitutively active EGFR ($EGFR^{CA}$) or to knockdown the expression of *dlg*, *scrib*, *lgl*, *Chc*, *shi*, or *AP-2α* (Figures 1D–1F, 1H–1J, S1B–S1E, and S1G–S1J). Flies also carried an $αtubGal80^{ts}$ transgene, which allows *GAL4* activity at 30°C, allowing gene overexpression or downregulation in a time-controlled manner.

Analysis of the tissue 4 days (4d) and 7 days (7d) after shift to 30°C allowed us to observe the progression of the phenotypes over time. Similar to what we previously showed (Papagiannouli et al., 2019), EGFR overactivation in CCs for 4d (either by forced expression of $EGFR^{CA}$ or by knockdown of Dlg- or CME-module components, hereafter called "EGFR overactivation") led to loss of spermatogonia and spermatocytes via apoptosis, visualized by visible gaps in the Vasa staining (Figures 1D–1F and S1B–S1E). TUNEL staining marked the dying GL, which appeared as red-blebs devoid of cell-type-specific markers, at more advanced stages of apoptosis (Figures 1H–1J and S1G–S1J). CCs clustered together, creating large "patches" marked by mCD8+ areas, in the absence of the germ cells they normally encapsulate (Figures 1D–1F and S1B–S1E). However, the underlying signal that mediates GCD upon EGFR overactivation in CCs remained so far uncharacterized.

Overactivation of EGFR in CCs leads to upregulation of JNK signaling in CCs and ROS oxidative stress in the neighboring GL

The JNK pathway is an evolutionarily conserved kinase cascade with an important role in stress-induced apoptosis and tumor progression (Herrera and Bach, 2021). In normal, physiological conditions, JNK signaling is activated in CySC and CCs via the JUN kinase Bsk, leading to the activation of the downstream effectors puckered (Puc) and matrix metalloprotease (Mmp1) (Brantley and Fuller, 2019; Chang et al., 2020; Herrera and Bach, 2018; 2021; Senos Demarco and Jones, 2019; Tang et al., 2017; Zohar-Fux et al., 2022). Here, basal JNK levels were observed in control testes by monitoring protein levels of Mmp1 (Figures S1K–S1M) and AP-1-responsive TRE elements fused to GFP (*puc::TRE-GFP*) (Chatterjee and Bohmann, 2012), with the latter reflecting *puc* expression levels in CCs (Figures 1G, 2A, 2E, S1F, S2A, S2F, and S2K). Overexpression of $EGFR^{CA}$ showed Mmp1 staining in CCs that clustered together after 4d and 7d of activation (Figures S1L and S1N). Quantification of corrected Mmp1 fluorescence confirmed the increased Mmp1 levels for both 4d and 7d (**$p < 0.01$ and ****$p < 0.0001$, respectively), providing the first link of JNK upregulation following EGFR overactivation in CCs. Yet, as the Mmp1 staining is challenging in the *Drosophila* testis, we focused our further analysis on *puc* expression, using *puc::TRE-GFP* as a readout for JNK signaling levels.

Overexpression of $EGFR^{CA}$ or knockdown of Dlg module or CME components using the *c587-GAL4* resulted in bright GFP staining reflecting *puc* expression in clusters of CCs that encapsulate TUNEL-positive germ cells (Figures 1H–1J and S1G–S1J), thereby linking JNK upregulation in CCs to the death of the germ cells they encapsulate. To confirm JNK upregulation in this context, we looked closer at *puc* expression upon EGFR overactivation in CCs (Figures 2A–2H and S2A–S2O). Quantification of corrected *puc::TRE-GFP* levels after 4d of *UAS* activation (Figure 2M) showed significant upregulation of JNK signaling levels. Each individual sample compared to the control showed a value of ****$p < 0.0001$. Similar results were also obtained when we quantified corrected *puc::TRE-GFP* levels after 7d (Figures S2K–S2P) of $EGFR^{CA}$ overexpression or *dlg*, *scrib*, *Chc*, *shi*, and *AP-2α* knockdowns in CCs. More precisely, $EGFR^{CA}$ overexpression and *dlg* knockdown showed a stable 3- and 2-fold increase in 4d and 7d activation, respectively. CME components showed 4- to 6-fold increase in 4d activation, after which levels dropped to a 2.5- to 3.5-fold increase (****$p < 0.0001$) compared to the control. Loss of *scrib* and *lgl* in CCs was accompanied by a 2-fold increase of *puc* levels after 4d (****$p < 0.0001$), which dropped to 1.5 (**$p < 0.01$) for *scrib* and to non-significant levels for *lgl* after 7d. The weaker phenotype observed upon loss of *lgl* was consistent with the weaker efficiency of the *UAS-lgl-RNAi* fly lines, matching our previous study (Papagiannouli et al., 2019). Importantly, knocking down *bsk* in CCs led to efficient reduction of *puc* expression levels (****$p < 0.0001$), thereby confirming the effectiveness of the *UAS-bsk-RNAi* transgene in downregulating JNK signaling in these cells (Figure 2M; column 2 vs. 1).

ROS are oxygen derivatives acting as signaling molecules in physiological levels to promote tissue regeneration or wound healing, whereas elevated levels can induce oxidative stress resulting in cellular damage and cell death (Lennicke and Cocheme, 2021; Schieber and Chandel, 2014; Sinenko et al., 2021). Previous studies have shown ROS activation in the GL of *Drosophila* testes (Senos Demarco and Jones, 2019; Tan et al., 2017). Staining of testes with dihydroethidium (DHE) to detect ROS levels showed increased levels of ROS in differentiating germ cells upon $EGFR^{CA}$ overexpression or knockdown of Dlg module and CME components in CCs (Figures 2J–2L and S2R–S2U), compared to the physiological levels of the control (Figures 2I and S2Q). Quantification of the DHE levels for each genotype after 4d of activation (Figure 2N) confirmed these observations. All genotypes showed significant upregulation of ROS levels (****$p < 0.0001$), while *lgl* knockdown showed significant but lower ROS activation (**$p < 0.01$). This latter observation is most likely due to the weaker efficiency of the *UAS-lgl-RNAi* fly lines (Papagiannouli et al., 2019).

Our results showed that EGFR overactivation in CCs (upon either forced expression of $EGFR^{CA}$ or loss of function of Dlg module or CME components) led to upregulation of JNK signaling in CCs and ROS in the neighboring germ cells prior to their death.

Lowering the levels of the JUN kinase *Basket* in CCs showed a partial rescue of GCD and reduction in ROS levels in the GL

Given the elevated JNK activity following EGFR upregulation in CCs, we tested if reducing JNK activation might rescue the GCD phenotype. To interfere with JNK signaling, we used a transgenic RNAi to knockdown *bsk* in the CCs, as this was shown to effectively downregulate *puc* expression in CCs (“*bsk*-rescue”) (Figure 2M; column 2). To control for possible effects of multiple *UAS* constructs limiting the effectiveness of the *GAL4* driver, control flies carried the same number of *UAS* transgenes using a *UAS-mCD8-GFP*. Because different *UAS* transgenes have different expression strengths, and the phenotype manifested is the result of the equilibrium of 2 *UAS* lines, rescue experiments were performed by shifting flies to 30°C for 4d and 7d.

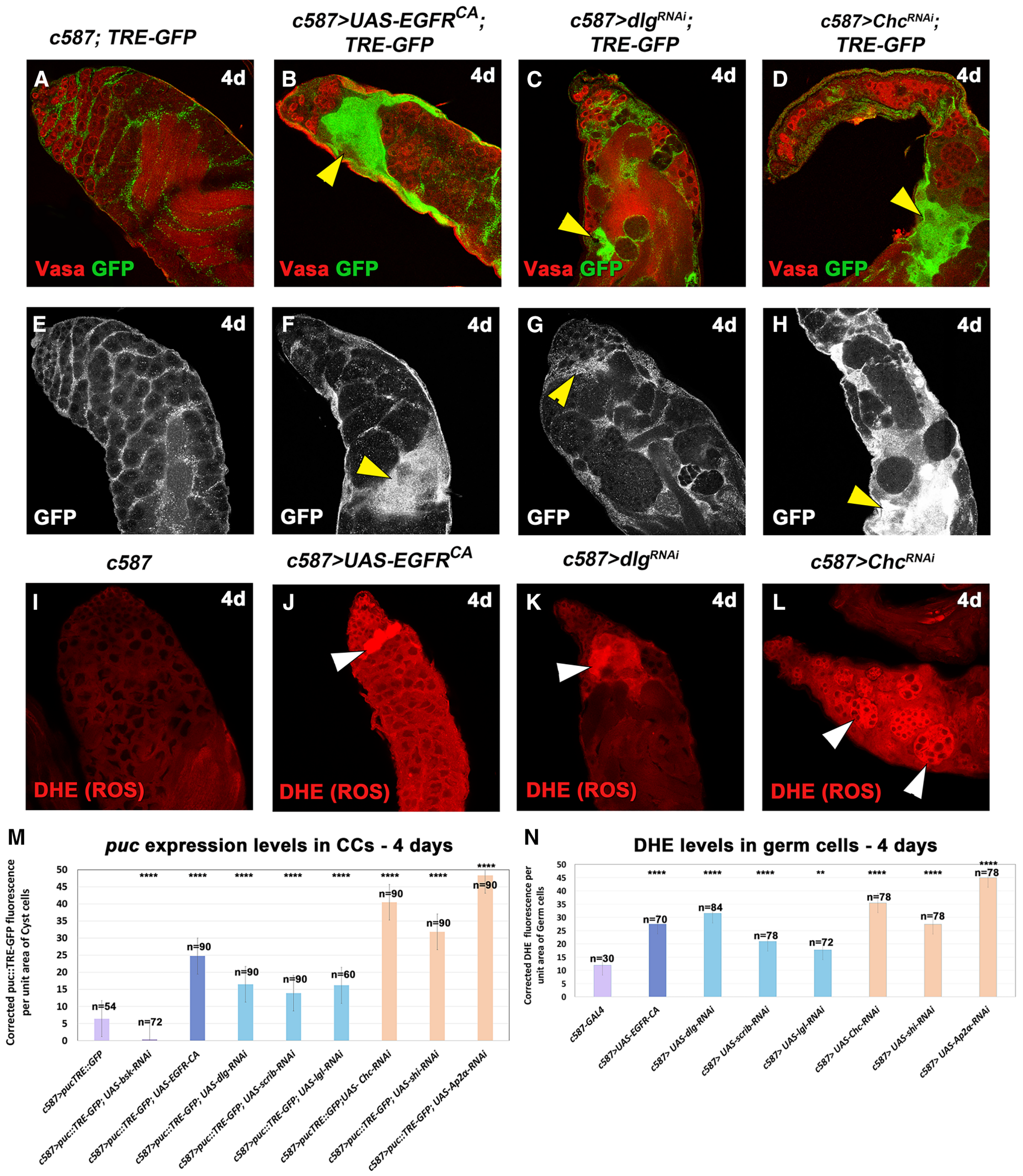

Figure 2. Overactivation of EGFR in CCs leads to increased levels of JNK signaling in the CCs and ROS in the GL

(A–H) *TRE-GFP* reflects expression levels of JNK reporter *puc* in CCs. (A–D) *TRE-GFP* (green; CCs); Vasa (red; GL). (E–H) *TRE-GFP* levels (white) in directly comparable raw images. Yellow arrowheads: regions of *puc::TRE* overactivation in CCs.

(I–L) DHE (red) reflects ROS activation in the GL. White arrowheads: representative areas of ROS activation in the GL.

(M and N) Quantification of corrected fluorescent *puc::TRE-GFP* levels in CCs and DHE levels in germ cells, respectively. Each individual sample was compared to control (error bars: standard error; ns: not significant; *$p < 0.05$, **$p < 0.01$; ***$p < 0.001$; ***$p < 0.0001$). Numbers (n) in each column represent sample size. Dotted plots in Figure S2V—S2W. Image frames (A–L): 225 μm.

Representative examples of the different phenotypic classes, reflecting the variability in strength and different degree in restoring normal structure and progression of GL differentiation of the rescued phenotypes in comparison to control flies (wild-type-like *c587> UAS-mCD8-GFP*), are shown in Figures 3 and S3. The observed phenotypes were classified into the following categories: (1) "no rescue" for testes mimicking the effect of acute $EGFR^{CA}$

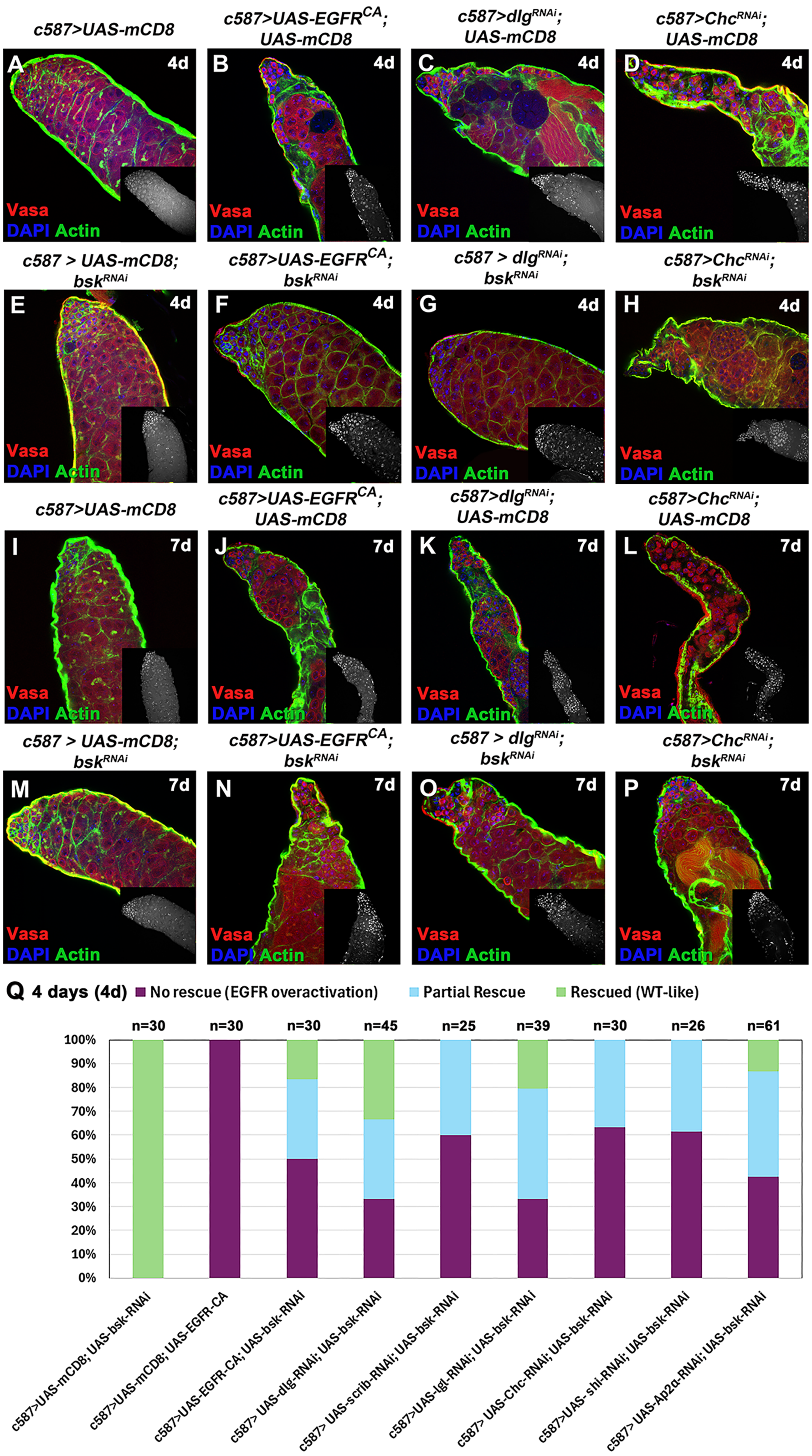

(legend on next page)

overexpression in CCs (Figures 3B–3D and S3B–S3E [4d]; Figures 3J–3L and S3L–S3O [7d]) with decreased number of germ cells and CC clustering together in patches devoid of germ cells; phenotypes here ranged from milder to stronger ones; (2) “partial rescue” for testes with densely packed differentiating germ cells (and minor germ cell loss if any), where encapsulation by CCs was largely restored but architecture and overall morphology appeared distorted or few CC clusters could still be observed (Figures 3H, 3N–3P, S3J, S3Q, and S3S–S3T). (3) “Rescued (wild-type-like)” for testes with restored spermatogonial and spermatocyte cysts (Figures 3F, 3G, S3G–S3I, and S3R).

Although a range of phenotypes resulted from our rescue strategy, the percentage of testes showing “no rescue” (mimicking the “EGFR overactivation” phenotype) was substantially reduced and represented 30%–62% of the overall testes scored after 4d rescue (Figure 3Q) and 0%–63% after 7d rescue (Figure S3U), compared to 100% penetrance before the rescue (Figures 3Q and S3U; column 2) (Papagiannouli et al., 2019). Conversely, the testes scored for full and partial rescue combined represented a minimum of ~40% after 4d and minimum of 53% after 7d, evidenced by a largely restored morphology with packed GL cysts.

Effective reduction of JNK signaling in these *bsk*-rescue experiments was confirmed by quantifying corrected *puc::TRE-GFP* fluorescence for each genotype for 4d activation (Figures 4A–4J). The results showed significant reduction in GFP fluorescence reflecting *puc* expression levels in *bsk*-rescued testes (Figure 4P), compared to the EGFR overactivation phenotypes (Figure 2M). We could observe a significant reduction in *puc* levels upon single knockdown of *bsk* in CCs compared to the control (Figure 4P; column 2 vs. 1; ****$p < 0.0001$) and a milder reduction of *puc* levels in the *bsk*-rescue experiments (Figure 4P; columns 3–5 vs. column 1; ****$p < 0.0001$).

Next, we investigated the levels of ROS in *bsk*-rescued testes after 4d activation (Figures 4K–4O). Knocking down *bsk* in CCs (1× *UAS* line) reduced ROS levels significantly (****$p < 0.0001$) compared to the control (*c587>UAS-mCD8*; 1× *UAS* line) (Figure 4Q; columns 2 vs. 1), showing that lowering JNK levels affects basal ROS levels in the GL. Comparing *bsk*-rescued testes (containing 2× *UAS* lines) to the control and the *mCD8-bsk-RNAi* control (2× *UAS* lines) showed a reduction in ROS levels (Figure 4Q; columns 3–5 vs. 1–2) compared to “EGFR overactivation” testes (Figure 2N). Yet this reduction was not sufficient to bring ROS down to control (physiological) levels (as in the case of *puc* levels; Figure 4P). To compare DHE levels in *bsk*-rescued vs. “EGFR overactivation” phenotypes (Figures 2N vs. 4Q), we normalized the control levels between the two datasets (because pictures for the quantifications were taken with a different laser). Comparison of the two datasets showed that ROS levels were reduced by 30% in *bsk*-rescued testes with $EGFR^{CA}$ overexpression, 20% in *dlg/bsk* double-knockdowns, and 43% in *Chc/bsk* double-knockdowns.

To test whether GCD was specifically reduced in the JNK-mediated rescue of EGFR overactivation phenotypes, we performed TUNEL assays after 2d (Figures S4A–S4H) and 4d (Figures S4I–S4P) of activation. Results showed that in early 2d activation, there is a small yet significant activation of GCD, with no significant reduction of GCD in *bsk*-mediated rescue (Figure S4H) at this stage. However, after 4d of activation, we observed a significantly stronger activation of GCD in EGFR overactivation phenotypes (Figure S4P) and significant reduction of GCD in *bsk*-mediated rescue (Figure S4H). We could therefore conclude that effective reduction of JNK levels in CCs experiencing increased levels of EGFR reduced JNK levels in CCs and ROS levels in the neighboring GL and GCD.

Feeding flies with antioxidant vitamin C reduces ROS levels in the GL, JNK levels in CCs, and GCD

Since our previous results have shown that reducing JNK signaling in CCs could lower ROS levels in the GL and prevent GCD, we tested whether the opposite was also true, i.e., whether reducing ROS levels in the germ cells not only prevented GCD but could also reduce JNK levels in the neighboring CCs. We used Vit.C, an antioxidant that has been proven effective in reducing levels of ROS oxidative stress previously (Senos Demarco and Jones, 2019). Flies overexpressing $EGFR^{CA}$ in CCs for 4d at 30°C were fed with Vit.C for the last 2 days prior to the dissection, and the phenotypes were subsequently analyzed (Figures 4R–4V, 5B, and 5J). Results showed that Vit.C reduced ROS significantly, to levels comparable to the physiological ones (*c587* control) (Figures 4T and 4U). Importantly, feeding flies with Vit.C led to significant reduction of *puc* levels in CCs, comparable to the reduction observed when *bsk* was depleted in the same context (*bsk*-rescue) (Figures 4R, 4S, and 4V; columns 3 and 4). These observations were also combined with a partial or full rescue of the “EGFR overexpression” phenotype, which reached

Figure 3. Knocking down the JUN kinase *bsk* in CCs can partially rescue the GCD phenotype

(A–P) Vasa (red; GL), DAPI (blue; nuclei), and actin stained with phalloidin (green channel; hub, CySCs, CCs, and GL fusome) also in flies containing the *mCD8-GFP* transgene (since the GFP is not shown here).

(Q) Quantifications of the different phenotypic classes accompanying each genotype, organized in the order of phenotypic strength. Image frames (A–P): 225 μm.

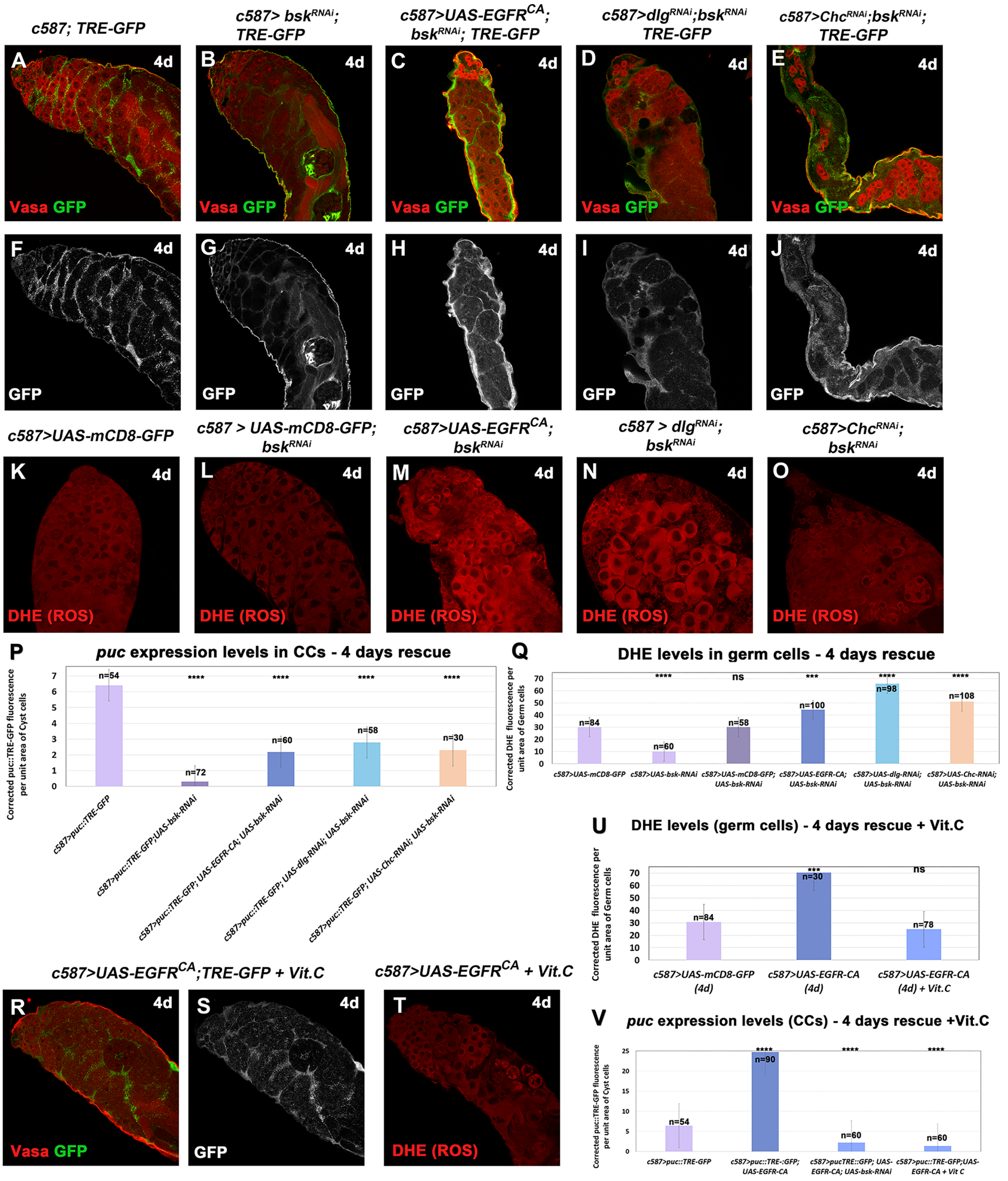

Figure 4. Knocking down the JUN kinase *bsk* lowers JNK signaling levels in CCs and ROS levels in the GL

(A–J, R, S) *TRE-GFP* reflects expression levels of JNK reporter *puc* in CCs. (A–E) *TRE-GFP* (green; CCs); Vasa (red; GL). (F–J, S) show the *TRE-GFP* levels only (white) in directly comparable raw images.

(K–O and T) DHE (red; GL) reflects ROS activation in the germ cells. Testes in (R–T) were treated with antioxidant vitamin C (Vit.C).

(P and V) Quantification of corrected fluorescent *puc::TRE-GFP* levels in CCs.

(Q and U) Quantification of corrected fluorescent ROS levels in germ cells. Each individual sample was compared to control (error bars: standard error; ns: not significant; $*p < 0.05$; $**p < 0.01$; $***p < 0.001$; $****p < 0.0001$). Numbers (n) in each column represent sample size. Dotted plots in Figures S5I–S5L. Image frames (A–O and R–T): 225 μm.

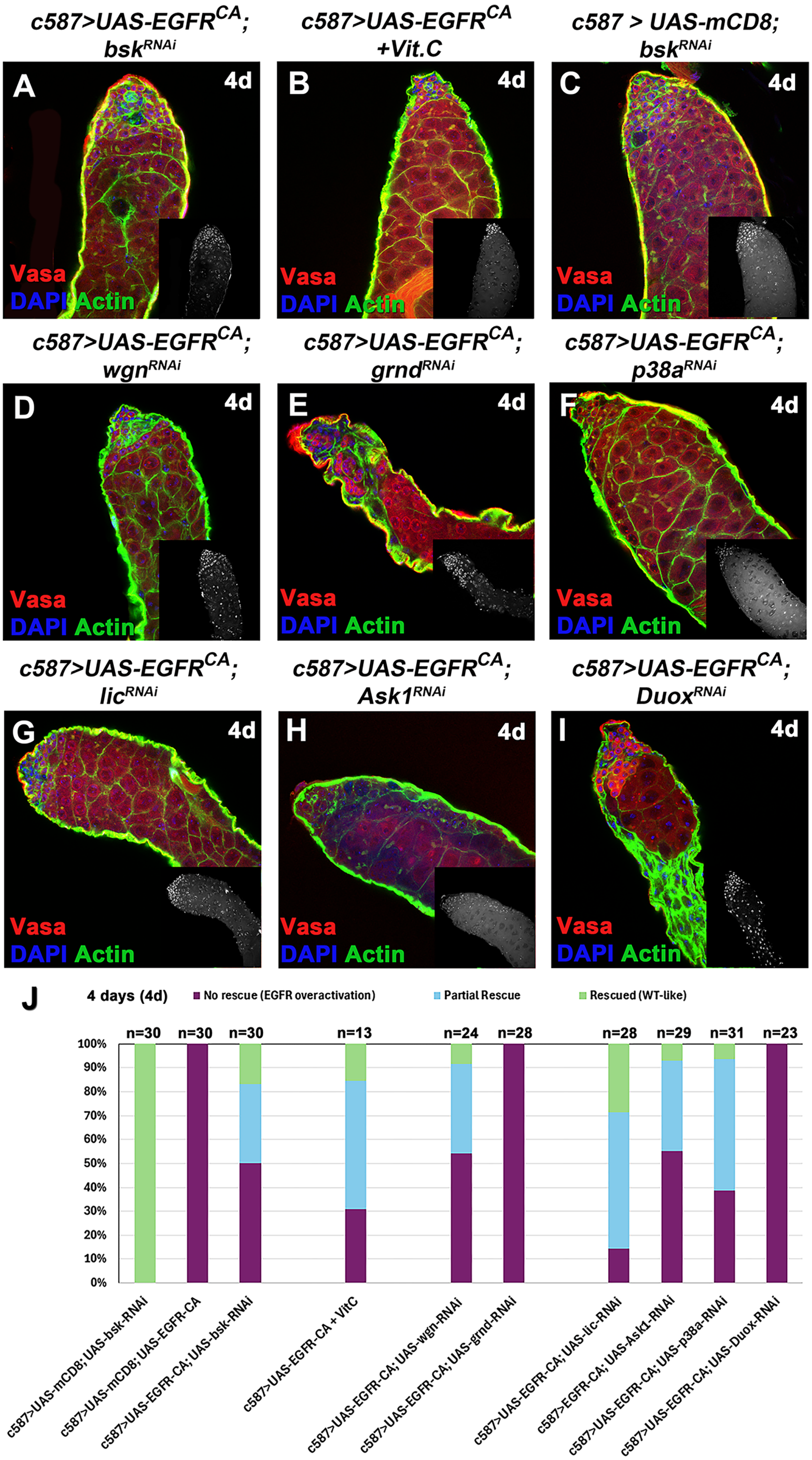

Figure 5. EGFR overactivation phenotypes can be partially rescued after treatment with antioxidant Vit.C or by knocking down the JNK receptor Wgn and p38 pathway components in CCs

(A–I) Vasa (red; GL), DAPI (blue; nuclei), and actin (green channel) also in flies containing the *mCD8-GFP* transgene (since the GFP is not shown here).

(J) Quantification of the different phenotypic classes accompanying each genotype, organized in the order of phenotypic strength. Image frames (A–I): 225 μm.

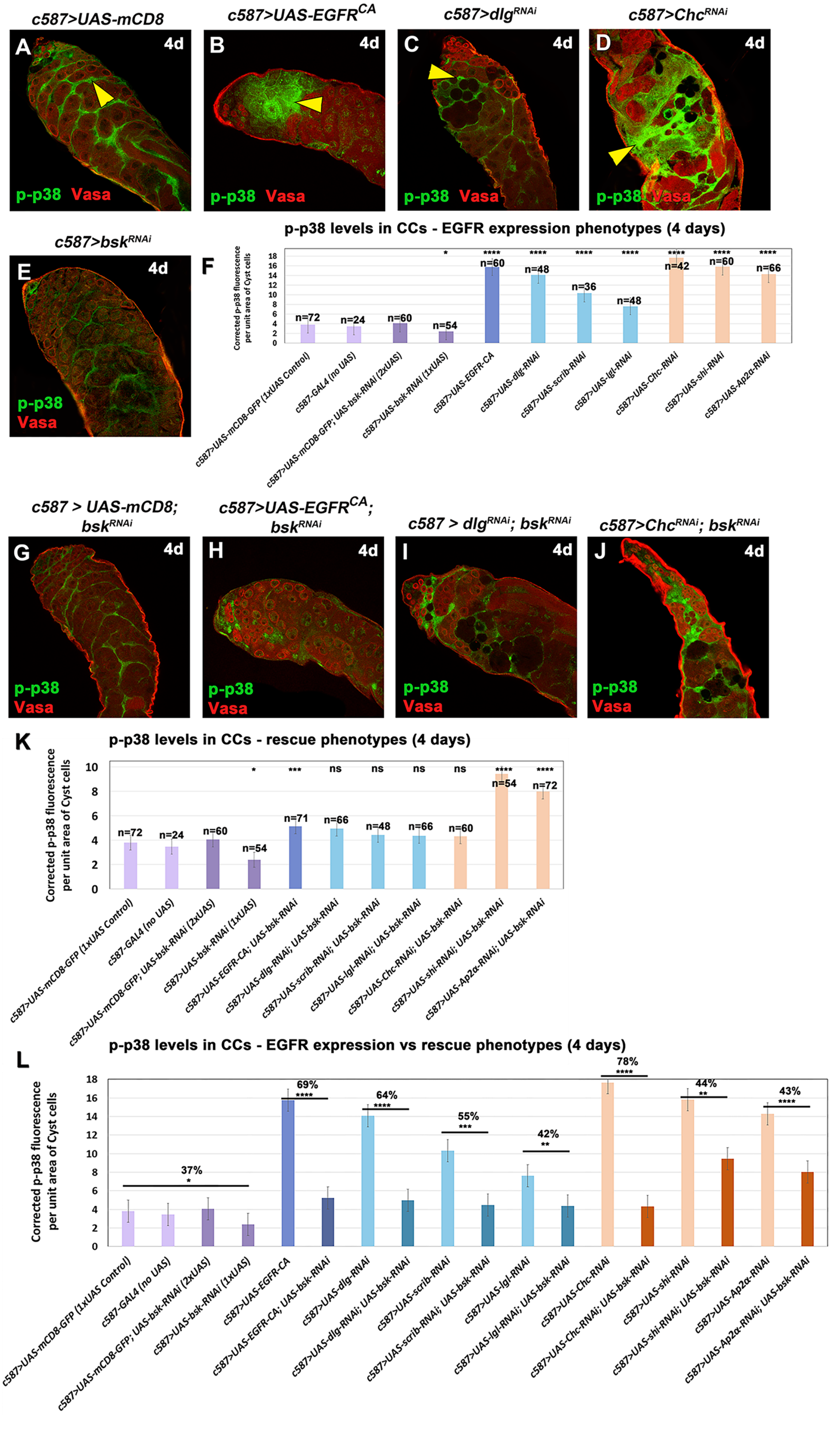

(legend on next page)

70% of the testes scored after 4d activation (Figures 5B and 5J; column 4) and 88% after 7d activation (Figures S5C and S5H; column 4). Interestingly, the Vit.C-rescue seemed to be more efficient than the *bsk*-rescue in the CCs (Figures 5J and S5H; columns 4 vs. 3 in both).

JNK receptor Wgn, apoptosis signal-regulating kinase 1, and p38 signaling are involved in CC-GL communication as a result of EGFR overactivation in CCs

To understand how JNK signaling is activated in CCs, we looked at both JNK TNF receptors present in *Drosophila*, Wgn, and Grnd (Colombani and Andersen, 2023). Knocking down *wgn* in CCs overexpressing $EGFR^{CA}$ partially rescued the EGFR overactivation phenotype (Figure 5D; column 5). Interestingly, the pattern of the three phenotypic classes ("no rescue," "partial rescue," "rescued") between *wgn*- and *bsk*-rescues was very similar (Figure 5J; compare column 5 vs. 3). On the other hand, knocking down *grnd* in CCs in the background of $EGFR^{CA}$ overexpression could not rescue the phenotype (Figures 5E and 5J; column 6).

Ask1 is a serine/threonine kinase, which senses ROS cells, and in response to diverse stresses activates the JNK and p38 MAPK pathways (Patel et al., 2019; Santabarbara-Ruiz et al., 2019). As not only JNK but also p38 signaling is involved in apoptotic responses, we investigated the involvement of key components of the Ask1-p38 cascade (Ask1, Licorne [Lic], p38a, and Duox) in CCs. Single knockdown of any of these genes for 4d did not have obvious defects in differentiating germ cells or CCs (Figures S5D–S5H; columns 7–10), same as already observed in the case of single *bsk* depletion (Figures 5C, 5J, and S5H; column 1 in both). Knockdown of *Ask1*, *lic*, or *p38a* in CCs overexpressing $EGFR^{CA}$ could rescue the EGFR overactivation phenotypes, with no gaps in the Vasa staining and restored encapsulation by the CCs (appearing as squamous thread-like green structures in confocal pictures) (Figures 5F–5H). Knockdown of *Duox* could not rescue the phenotype with CCs clustering phenotype (Figure 5I). Quantifying the different phenotypic classes revealed that testes scored as partially or fully rescued represented 86% in the case of *lic*, 45% for *Ask1*, 61% for *p38a*, and 0% for *Duox* (Figure 5J).

To establish a more direct link between EGFR overactivation, GCD, and the p38 pathway, the levels of phosphorylated p38 were investigated using an anti-phospho-p38 (p-p38) antibody that should recognize activated p38a and p38b but not p38c (Patel et al., 2019). In control testes, p-p38 was clearly decorating CC nuclei but was also present in the CC cytoplasm (Figures 6A and S6A). Immunostaining of adult testes overexpressing $EGFR^{CA}$ or depletion of *dlg*, *scrib*, *lgl*, *Chc*, *shi*, or *AP-2α* in CCs showed significant increase of p-p38 levels (****p < 0,0001) (Figure 6F; columns 5–11) in the nuclei of CCs that cluster together devoid of the germ cells they normally encapsulate (Figures 6B–6D and S6B–S6E) compared to the control (1× *UAS* or no *UAS*; Figures 6A and 6F; columns 1, 2). In CCs depleted of *bsk* only, p-p38 levels were significantly reduced (*$p < 0.05$) (Figures 6E and 6F; column 4), while in the control containing two *UAS* lines (*UAS-mCD8*; *UAS-bsk-RNAi*) p-p38 levels were almost comparable to physiological levels (Figures 6G and 6F; column 3 vs. 1–2). p-p38 levels dropped to non-significant (ns) levels (Figure 6K; columns 6–9) in rescue experiments with double knockdowns of *bsk* with *dlg*, *scrib*, *lgl*, or *Chc* (Figures 6I, 6J, S6G, and S6H), a reduction ranging between 42% and 78% (Figure 6L). In the case of $EGFR^{CA}$, *shi*, or *AP-2α* (Figures 6H, S6I, and S6J), rescued testes showed a 43%–69% reduction in p-p38 levels (Figure 6L) even though these levels were still statistically significant (***$p < 0.001$ or ****$p < 0.0001$) (Figure 6K; columns 5, 10, and 11) compared to the control (*c587>UAS-mCD8*). Therefore, JNK signaling regulates p38 MAPK signaling, since modifying *bsk* levels affected p38 phosphorylation and activation in CCs.

The GTPase Rab35 is required in CCs to regulate JNK, ROS, and EGFR response following EGFR overactivation

To investigate some of the more downstream regulators of CC-GL communication following the upregulation of the EGFR in CCs, we investigated the role of Rab35. Rab35 is a GTPase of the recycling endosome involved in plasma membrane transport, polarized trafficking, phagocytosis,

Figure 6. Levels of phosphorylated MAPK p38 increase in CCs experiencing EGFR overactivation and in response to JNK-derived cues

(A–E and G–J) Vasa (red; GL), DAPI (blue; nuclei), and p-p38 (green; CCs and nuclei), also in flies containing the *mCD8-GFP* transgene (since the GFP is not shown here). Yellow arrowheads point at CCs with high levels of p-p38. Small inset pictures show the p-p38 staining only.

(F and K) Quantification of corrected fluorescent p-p38 levels in CCs (and their nuclei) of indicated genotypes with "EGFR overactivation" (F) and *bsk*-rescue (K) background. For statistics in (F and K) each individual sample was compared to the *c587>UAS-mCD8* control.

(L) Combined quantifications from (F and K), compare p-p38 levels in "EGFR overactivation" vs. *bsk*-rescue phenotypes. For statistics, "EGFR overactivation" genotypes were compared to *bsk*-rescues, while numbers represent % of p-p38 reduction (error bars: standard error; ns: not significant; *$p < 0.05$; **$p < 0.001$; ***$p < 0.001$; ****$p < 0.0001$). Numbers (n) in each column represent sample size. Dotted plots in Figures S6K–S6M. Image frames (A–Q): 225 μm.

and exosome secretion (Ochi et al., 2022; Shim et al., 2010). Use of a Rab35-GFP protein-tag in control testes showed Rab35 localization in CCs and spermatogonia (Figures S6N–S6N′). Rab35 was still present in CCs overexpressing *EGFR*^{CA} or upon loss of *dlg* or *Chc* (Figures S6P–S6R′). However, Rab35 was not any more visible after knocking down *Rab35* in CCs, an effect that was not associated with other visible defects in CCs and differentiating germ cells (Figures S6O–S6O′).

Knocking down *Rab35* in CCs for 4d, in the background of *EGFR*^{CA} overexpression or loss of *dlg* and *Chc* (Figures 7A–7E), led to a partial rescue of the EGFR overactivation phenotype, although this was less efficient compared to the *bsk*-rescues (Figures 7M [columns 4–6 vs. 3] and 3Q]. When we compared *Rab35*-rescued vs. *EGFR*^{CA}-overexpressing testes, we observed significant reduction in *puc* expression levels in CCs (Figure 7N; column 5 vs. 2) and ROS levels in germ cells (Figure 7O; column 5 vs. 2). Yet, when we compared *bsk*-rescued vs. *Rab35*-rescued testes, reduction of *puc* levels was more significant in *bsk*-rescued testes (Figure 7N; column 5 vs. 3), while reduction of ROS levels was at similar comparable levels between *bsk*- and *Rab35*-rescued testes (Figure 7O; column 3 vs. 5).

We then looked at whether Rab35 could regulate the localization of the EGFR and JNK receptors in CCs, using a GFP-tagged Wgn transgene and a genomic EGFR-GFP construct (Figure S7). Levels of Wgn-GFP and EGFR-GFP localization in CCs were comparable in *Rab35-RNAi* testes compared to controls (Figures S7H [columns 3 vs. 1 and 2] and S7O [column 2 vs. 1]). As expected, we saw strong localization of Wgn-GFP and EGFR-GFP in EGFR overactivation phenotypes (Figures S7H [columns 4, 6, and 8] and S7O [column 3, 5, and 6]) and significant increase compared to controls and Rab35-RNAi testes. Interestingly, we observed a much stronger localization of Wgn-GFP upon *dlg* knockdown in CCs (Figure S7H; column 6 vs. 4 and 8) and a much stronger localization of EGFR-GFP upon *Chc* knockdown in CCs (Figure S7; column 6 vs. 3 and 5), which could potentially reflect a difference in the function of polarity vs. CME components. Importantly, in *Rab35*-mediated rescues, we observed significant reduction in both Wgn and EGFR localization (Figure S7H [columns 4, 5, 6, and –7] and S7O [columns 3 and 4]), linking Rab35 function to both JNK and EGFR signaling receptors. Thus, Rab35 emerged as one of the EGFR and JNK downstream effector genes that mediates CC-GL reciprocal communication and transduction of the death signal.

DISCUSSION

Short-range communication between closely apposed cells is critical for building functional tissues and organs and maintaining homeostasis. The *Drosophila* testis provides an excellent system to study *in vivo* how closely apposed cell types reciprocally communicate and coordinate their co-differentiation (Herrera et al., 2021; Zoller and Schulz, 2012). Our results show that the cortical polarity proteins Dlg, Scrib, Lgl, and CME fine-tune EGFR signaling levels and thereby influence the network of JNK, p38, and ROS signaling cross-regulation between the CCs and the differentiating germ cells. As in previous studies (Chang et al., 2019, 2020; Herrera et al., 2021; Herrera and Bach, 2018; La Marca et al., 2019; Senos Demarco and Jones, 2019; Tan et al., 2017), we observe basal (physiological) levels of JNK in CySCs and CCs and ROS in the GL, although we primarily focused on the differentiating CCs and germ cells where our phenotypes are manifested. Our findings have shown that depletion of *dlg*, *scrib*, *lgl*, or CME components in CCs leads to EGFR overactivation, accompanied by upregulation of JNK and p38 in CCs, and ROS in germ cells destined to die (Figure 7P). GCD could be reversed by acting either in the CCs through reduction in JNK signaling levels or directly in the GL by reducing ROS levels. More precisely, knocking down *bsk* in CCs reduced intrinsic levels of JNK and p38 as well as ROS levels in the neighboring GL. Yet, a reduction of ROS levels in germ cells by feeding flies with Vit.C seemed to be more effective in reducing JNK levels in CCs (compared to the reverse), suggesting that oxidative stress could potentially have a more instructive role in directing CC behavior besides triggering GL apoptosis. This could be explained by GL-derived ROS acting directly on the EGFR signaling pathway, as suggested previously (Senos Demarco and Jones, 2019) and because the EGFR upstream of the signaling cascade in CCs can confer a more efficient rescue.

In tissues that harbor stem cell linages, JNK signaling is activated in a variety of stress responses with an instructive role in regulating the balance between homeostasis and tissue regeneration vs. degeneration, aging, and death (Gan et al., 2021; Herrera et al., 2021). In *Drosophila* testes undergoing chronical stressful situations, JNK signaling seems to confer robustness by permitting Bam+/spermatogonia to dedifferentiate and renew the pool of GSCs (Herrera and Bach, 2018). Yet, stress linked to protein starvation leads to death of spermatogonia, by upregulating Spichthyin (Spict) in the encapsulating CCs (Chiang et al., 2017), which together with the lysosome promote spermatogonial phagocytic clearance. Similarly, germ cells that initiate the apoptotic pathway are eventually eliminated through the phagocytic action of JNK-upregulated CCs who extend their membranes into the dying germ cells (Zohar-Fux et al., 2022), which is very similar to our observations. In this study, we show a direct correlation between JNK upregulation and non-autonomous GCD, as TUNEL+ germ cells that turn on the apoptotic pathway are tightly wrapped by

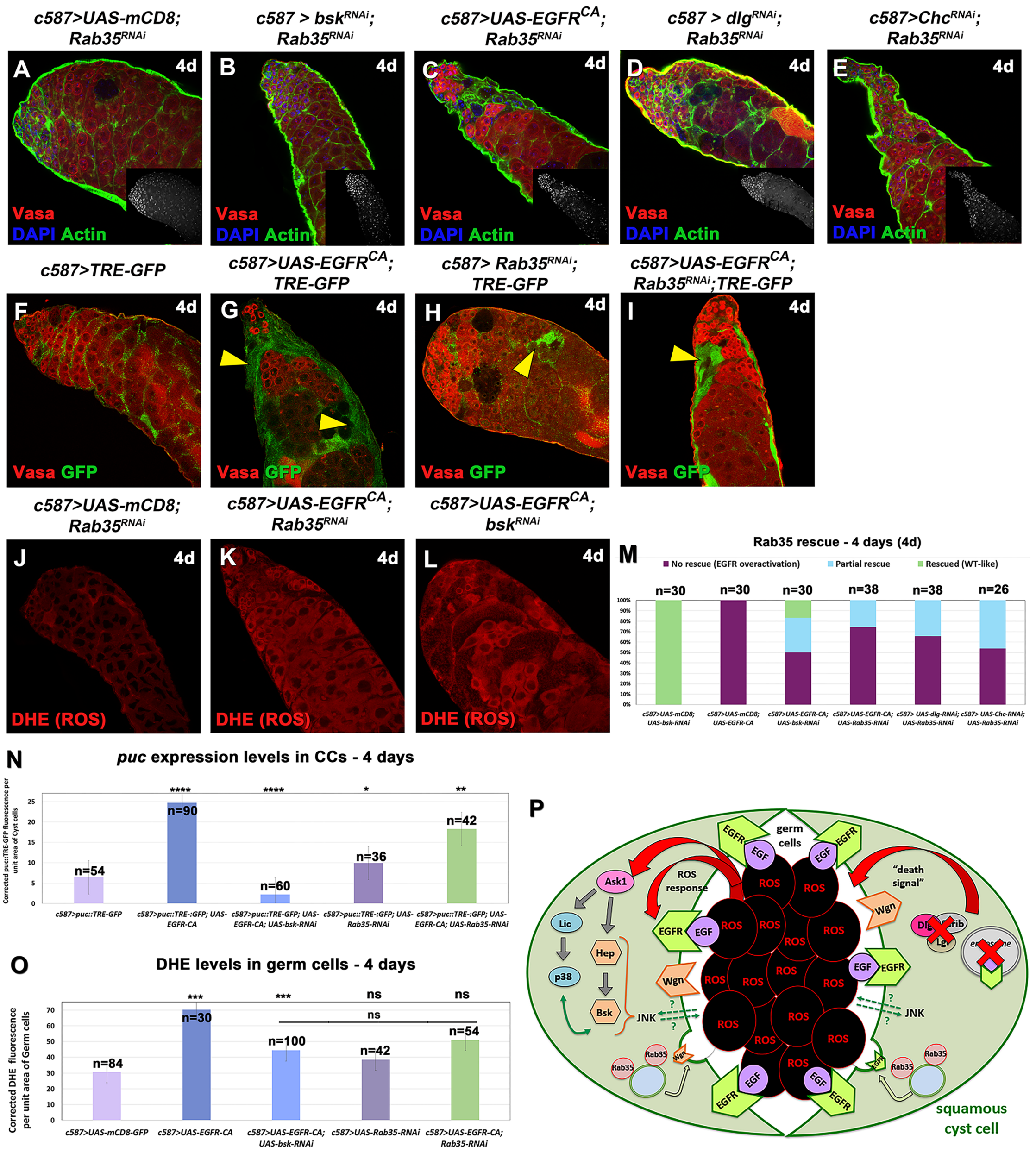

Figure 7. Knocking down the GTPase *Rab35* lowers JNK signaling levels in CCs and ROS levels in the GL

(A–E) Vasa (red; GL), DAPI (blue; nuclei), and Actin (green channel) also in flies containing the *mCD8-GFP* transgene (since the GFP is not shown here).

(F–I) *TRE-GFP* (green; CCs) reflects expression levels of JNK reporter *puc* in CCs; Vasa (red). Yellow arrowheads: regions of increased *puc::TRE* expression.

(J–L) DHE levels reflect ROS activation in the germ cells (red; GL).

(M) Quantification of the different phenotypic classes accompanying each genotype, organized in the order of phenotypic strength.

(N and O) Quantification of corrected fluorescent *puc::TRE-GFP* levels in CCs and ROS levels in germ cells, respectively. *TRE-GFP* reflects expression levels of JNK reporter *puc* in CCs. Each individual sample was compared to control (error bars: standard error; ns: not significant; $*p < 0.05$; $**p < 0.01$; $***p < 0.001$; $***p < 0.0001$). Numbers (n) in each column represent sample size. Dotted plots in Figures S7P and S7Q. Image frames (A–L): 225 μm.

(P) Model of JNK, p38, and ROS interplay in CC-GL cysts upon EGFR overactivation. Loss of Dlg-module or CME components in CCs upregulates EGFR signaling that sends a "death signal" to the GL (black) that activates ROS. JNK signaling, Rab35, and/or yet another

(legend continued on next page)

CCs with strong JNK-upregulation upon loss of the basal polarity Dlg/Scrib/Lgl complex and CME components. A similar mechanism of JNK upregulation that drives GCD has been described upon loss of the apical polarity Baz/aPKC/Par6 complex in CCs, with the difference that GCD was activated only in spermatocytes and not in spermatogonia (Brantley and Fuller, 2019). However, the underlying reason that drives GCD in different germ cells in basal vs. apical components is currently unknown. A reciprocal signal, where cells with phagocytic potential and dying cells signal to each other has been described in *Drosophila* ovaries (Serizier et al., 2022). There, the phagocytic receptor Draper in epithelial follicle cells induces non-autonomous cell death in the neighboring nurse cells through a JNK-dependent mechanism. The MAPK p38 has been also implicated in phagocytic encapsulation of bacterial pathogens, as an immune response to better tolerate bacterial infections (Shinzawa et al., 2009). Although our data show a direct correlation between JNK and p38 activity in CCs upon EGFR overactivation, whether p38 could also contribute to the phagocytic clearance of the dying GL we observed would require further investigation.

The EGFR signaling pathway regulates and co-ordinates a great number of fundamental functions in the *Drosophila* testis. Early studies have shown its importance in GL differentiation by directing the TA spermatogonial divisions, followed by the transition to the pre-meiotic spermatocyte program as EGFR signaling levels increase (Hudson et al., 2013; Sarkar et al., 2007; and references therein). The encapsulation of the GL by the CCs is also controlled by the EGFR, which regulates the activity of the downstream effector Rac1 that promotes the growth of CCs around the germ cells and the encapsulation strength by counteracting the function of Rho (Sarkar et al., 2007). A recent study highlighted the role of EGFR signaling in keeping the balance of CySC maintenance vs. CC differentiation, through regulation of autophagy-induced lipid breakdown (Senos Demarco and Jones et al., 2020). In CySCs, EGFR signaling stimulates autophagy through an AP-1-/Fos-mediated transcription of autophagy-related genes. Early CCs moving away from the hub suppress lipophagy via TOR to allow the CySC-to-CC fate switch to occur, while defective autophagy expands the CySC population in testes. On the other hand, ablation of CySCs activates the otherwise quiescent hub cells to turn on the EGFR signaling and resume the proliferation capacity of hub cells that can *trans*-differentiate and replenish CySCs (Greenspan et al., 2022). Thus, the EGFR plays an important role not only in promoting the GL differentiation and survival but also in safeguarding the proper fate balance within the somatic lineage (hub, CySCs, and CCs). Taken together, the EGFR emerges as an upstream master regulator of testis spermatogenesis in normal and stressful conditions, with the basal polarity Dlg/Scrib/Lgl complex and CME having an instructive role in EGFR-mediated functional homeostasis that is also intimately linked to JNK, p38, and ROS signaling.

ROS can activate JNK and p38 through a positive feedback loop and thereby regulate the balance between apoptosis and autophagy vs. cell competition, immunity, and stem cell and tissue regeneration in many tissues and organs across species (Binh et al., 2022; Diwanji and Bergmann, 2020; Kucinski et al., 2017; Santabarbara-Ruiz et al., 2015). Increased levels of ROS can propagate paracrine signals that are sensed by neighboring healthy cells, which involve activation of Ask1 and subsequent phosphorylation and activation of JNK and p38 (Esteban-Collado et al., 2023; Serras, 2022). In the mammalian testes, delicate levels of ROS mediate a positive feedback loop that sustains spermatogonial stem cell self-renewal by activating the MAPK p38 (Morimoto et al., 2019). In the *Drosophila* testis, upregulation of ROS levels in the GL have already been observed upon disruption of the antioxidant response of Keap1 (Kelch-like ECH-associated protein1)/Nrf2 (NF-E2-related factor 2) or mitochondrial fission by Drp1 (dynamin-related protein 1) (Senos Demarco and Jones, 2019; Tan et al., 2017). In both cases, increased levels of ROS result in loss of GSCs (and spermatogonia in the case of *Drp1* loss) and upregulation of the EGF-ligand Spitz, which overactivates the EGFR signaling pathway in the neighboring CCs. Our findings show that elevated levels of ROS in the GL can also result by directly modifying EGFR signaling levels in the CCs, like the ones observed upon loss of the Dlg-polarity and CME components or forced expression of EGFR in CCs, all leading to death of both spermatogonia and spermatocytes (Papagiannouli et al., 2019). We also saw that upregulation of EGFR in the CCs and ROS signaling in the GL is linked to JNK and p38 activation in CCs. Importantly, our rescue experiments support a model of reciprocal CC-GL communication between JNK/p38 and ROS signaling that controls GL survival, since suppressing *bsk* reduces p38 and ROS levels, while antioxidant treatment reduces ROS and normalizes JNK levels at the same time. Only few studies so far link the Dlg/Scrib/Lgl module to ROS oxidative stress, besides neoplastic tumors (Bunker et al.,

unidentified molecule mediate ROS activation. ROS responds back to the CCs by sensitizing Ask1 to activate the JNK and p38 MAPK signaling cascades, which signal back to the germ cells or by acting directly on EGFR signaling (red arrows). Downstream of the JNK/p38 MAPK cascade, Rab35 becomes activated (dashed green arrow), which contributes to the delivery of "death signal" at the CC-GL interface and regulates EGFR recycling in the membrane. Wgn is involved in activating JNK in CCs via Rab35.

2015). For example, Scrib regulates ROS levels and autophagy in mouse intestinal stem and epithelial cells, which is deregulated in inflammatory bowel disease (Sun et al., 2023). Moreover, loss of function of Dlg and Rab5 endocytosis is associated with ROS activation in fly nephrocytes (the equivalent to human podocytes) that affects slit-diaphragm integrity (Xi et al., 2024). Along these lines, we favor a model where EGFR/Ras/MAPK, JNK/p38, and ROS are part of a bidirectional feedback mechanism, in which EGFR upregulation can be both the cause and/or the consequence of ROS activation in the GL, while Dlg/Scrib/Lgl and CME act as guardians of this signaling homeostasis.

Our results have also indicated the importance of the TNF-α receptor Wgn in CCs experiencing EGFR overactivation, while Grnd seems not to be part of this signaling wiring. Given the fact that knockdown of *wgn* in CCs could reverse GCD (resulting from EGFR overactivation) in a rescue pattern and efficiency that was comparable to that of *bsk*- and *Ask1*-rescue, let us think that Wgn and Ask1 could be the "messengers" of the ROS-GL-mediated signal inside the CCs to activate JNK signaling. The two *Drosophila* TNF-α receptors, Wgn and Grnd, seem to have common functions albeit following a context-dependent mode of action within the cells they are functionally active. Egr/TNF-α can bind Wgn with an affinity that is three times weaker compared to that of Grnd (Palmerini et al., 2021), which could explain why in our system Wgn is involved in JNK activation, as a result of ROS overactivation in germ cells. Our hypothesis is further reinforced by studies showing that Wgn favors a more "local," close-range function (e.g., in neurons that rely on a local source of Egr) vs. Grnd that seems to be involved in more systemic stress responses. Along this line, it was previously shown that initiation of reproduction in *Drosophila* males activates the release of Egr from the smooth muscle sheath that surrounds the *Drosophila* testis to activate JNK signaling through Grnd. In this case, Grnd increases JNK in CCs at levels that are significant ($*p < 0.05$) (as shown by *puc::TRE-GFP*) (Chang et al., 2019) but not as high as in our experimental context ($****p < 0.001$) that is mediated by Wgn. Similarly, the Egr(smooth muscle)/Grnd(CCs) pathway is also activated upon protein starvation to promote the recovery of CySCs upon protein refeeding (Chang et al., 2020). Interestingly, Wgn has been shown to function in unconventional Egr-independent ways by becoming internalized in intracellular vesicles to regulate tracheal development (Letizia et al., 2023). Wgn has also been implicated in suppressing autophagy-dependent lipolysis in the *Drosophila* gut enterocytes to maintain homeostasis independently of Egr (Loudhaief et al., 2023), a function that in the *Drosophila* testis has been linked to target of rapamycin (TOR), which counteracts the function of EGFR/AP-1 in promoting lipophagy in early CCs (Senos Demarco and Jones, 2020; Senos Demarco et al., 2020). Therefore, we cannot exclude the possibility for Wgn to act in the *Drosophila* testis in an Egr-independent way that rescues the GCD phenotype in parallel to interfering with lipophagy.

We further showed that knockdown of the GTPase Rab35 could rescue testes in different degrees downstream of EGFR and JNK overactivation in CCs. Rab35 is a multifunctional protein of the recycling endosome, involved in membrane and endocytic trafficking that affects cytokinesis, cell adhesion, exosome release, and axon elongation (Allaire et al., 2013; Klinkert and Echard, 2016; Kouranti et al., 2006). Rab35 controls several aspects of apicobasal polarity with subcellular precision by regulating actin and microtubule dynamics as well as membrane PtdIns(4,5)P2 homeostasis (Francis et al., 2022; Ochi et al., 2022). By affecting actin and microtubule remodeling, Rab35 has been shown to mediate the transport of Cdc42 and Rac1 to the plasma membranes of filopodia-like protrusions involved in phagocytosis in *Drosophila*. Loss of Rab35 could rescue spermatocyte GCD when the apical Baz/aPKC/Par6 complex is disrupted in *Drosophila* testis, in line with our observations when the basolateral Dlg/Scrib/Lgl complex is depleted in CCs (Brantley and Fuller, 2019). Our data show that Rab35 acts downstream of EGFR and JNK signaling pathways by regulating their receptors in CCs and therefore mediates the death signal from the CCs to the GLs via an exocytosis-related mechanism or alternatively by membrane-targeting of the EGFR and Wgn receptors to the CC membranes and CC-GL interface.

This study highlights a bidirectional feedback mechanism that underlies CC-GL reciprocal communication that controls the signaling strength among EGFR, JNK, p38, and ROS within the *Drosophila* testis cysts. Cortical polarity components Dlg/Scrib/Lgl and CME play a pivotal role in maintaining the signaling output and physiological equilibrium that enables GL differentiation and spermatogenesis to proceed without problems. Even more, this work provides an important example on how polarity components cooperate with cellular trafficking and signaling mechanisms cell-intrinsically and across cells to build functional tissues and organs. It also provides a paradigm of how tissues can employ existing cellular networks and switch their physiological function to one that promotes cell death upon a certain threshold, when regeneration or alleviating the side effects of stress responses has failed and cannot no longer be reversed.

METHODS

Fly stocks and husbandry

Fly stocks used are described in FlyBase and listed in "supplemental methods". *UAS-gene*^{RNAi} stocks are referred

in the text as $gene^{RNAi}$. Flies also carried an *αtubGal80*^{ts} transgene, which blocks *GAL4* activity at 18°C but activates *GAL4* activity at 30°C, allowing overexpression or downregulation of gene function in a time-controlled manner, after normal testis anatomy had been set up. Crosses were raised at 18°C until adult flies hatched. Then males with the correct genotype were shifted at 30°C for 2, 4, or 7 days depending on the experimental needs. Lab work with *Drosophila melanogaster* (as a non-regulated, non-protected animal species in UK) is approved and oversighted by the University of Kent Animal Welfare Ethical Review Body (AWERB), and the University of Greenwich Biological & Genetically Modified Materials Safety (BGMS) Committee.

Immunofluorescence staining and microscopy

Whole-mount testes immunostainings, including TUNEL assays, were performed as previously described (Papagiannouli et al., 2019). A detailed ROS staining protocol, statistics, and list of antibodies and fly stocks can be found in "supplemental methods". Confocal images were obtained with a Zeiss/LSM880, processed with Adobe Photoshop 2025 and quantified with FiJi/ImageJ following "Corrected Total Cell Fluorescence". In Figures 2, 4, 5, 6, and 7, numbers (n) in each column represent sample size.

RESOURCE AVAILABILITY

Lead contact

Requests for further information and resources should be directed to and will be fulfilled by the lead contact, Dr. Fani Papagiannouli (f.papagiannouli-227@kent.ac.uk).

Materials availability

This study did not generate new unique reagents. All reagents used are listed in the supplemental methods.

Data and code availability

Microscopy data and additional information required to reanalyze the data reported here can be shared by the lead contact upon request.

ACKNOWLEDGMENTS

We thank the generous *Drosophila* community for reagents (David Bilder, Margaret Fuller, and Bassem Hassan), DSHB, VDRC, and BDSC Centers. We are grateful to Parthive Patel for sharing reagents on the p38 pathway and valuable feedback on our work, and Simon Richardson and Susan Shorter for the confocal microscope. Apologies to all whose work was not sited due to space limitations. This work was supported by Medway School of Pharmacy funding to F.P. and a PhD fellowship to M.A., The Royal Society Grant (RGS\R1\221141), University of Greenwich ECR Seed Funding to F.P., and 3MT awards to M.A.

AUTHOR CONTRIBUTIONS

M.A. co-designed, performed, interpreted experiments, assisted with writing the paper, and supported the study with her 3MT awards. F.P. designed, performed, interpreted experiments, wrote the paper, and obtained funding to support the study.

DECLARATION OF INTERESTS

The authors declare no competing interests.

SUPPLEMENTAL INFORMATION

Supplemental information can be found online at https://doi.org/10.1016/j.stemcr.2025.102676.

Received: June 29, 2024
Revised: September 15, 2025
Accepted: September 16, 2025
Published: October 16, 2025

REFERENCES

Allaire, P.D., Seyed Sadr, M., Chaineau, M., Seyed Sadr, E., Konefal, S., Fotouhi, M., Maret, D., Ritter, B., Del Maestro, R.F., and McPherson, P.S. (2013). Interplay between Rab35 and Arf6 controls cargo recycling to coordinate cell adhesion and migration. J. Cell Sci. *126*, 722–731. https://doi.org/10.1242/jcs.112375.

Binh, T.D., Nguyen, Y.D.H., Pham, T.L.A., Komori, K., Nguyen, T.Q. C., Taninaka, M., and Kamei, K. (2022). Dysfunction of lipid storage droplet-2 suppresses endoreplication and induces JNK pathway-mediated apoptotic cell death in Drosophila salivary glands. Sci. Rep. *12*, 4302. https://doi.org/10.1038/s41598-022-08299-6.

Brantley, S.E., and Fuller, M.T. (2019). Somatic support cells regulate germ cell survival through the Baz/aPKC/Par6 complex. Development *146*, dev169342. https://doi.org/10.1242/dev.169342.

Bunker, B.D., Nellimoottil, T.T., Boileau, R.M., Classen, A.K., and Bilder, D. (2015). The transcriptional response to tumorigenic polarity loss in Drosophila. eLife *4*, e03189. https://doi.org/10.7554/eLife.03189.

Chang, Y.C., Tu, H., Chen, J.Y., Chang, C.C., Yang, S.Y., and Pi, H. (2019). Reproduction disrupts stem cell homeostasis in testes of aged male Drosophila via an induced microenvironment. PLoS Genet. *15*, e1008062. https://doi.org/10.1371/journal.pgen.1008062.

Chang, Y.C., Tu, H., Huang, T.W., Xu, B.W., and Pi, H. (2020). Upregulated TNF/Eiger signaling mediates stem cell recovery and tissue homeostasis during nutrient resupply in Drosophila testis. Sci. Rep. *10*, 11674. https://doi.org/10.1038/s41598-020-68313-7.

Chatterjee, N., and Bohmann, D. (2012). A versatile PhiC31 based reporter system for measuring AP-1 and Nrf2 signaling in Drosophila and in tissue culture. PLoS One *7*, e34063. https://doi.org/10.1371/journal.pone.0034063.

Chiang, A.C.Y., Yang, H., and Yamashita, Y.M. (2017). spict, a cyst cell-specific gene, regulates starvation-induced spermatogonial cell death in the Drosophila testis. Sci. Rep. *7*, 40245. https://doi.org/10.1038/srep40245.

Colombani, J., and Andersen, D.S. (2023). Drosophila TNF/TNFRs: At the crossroad between metabolism, immunity, and tissue homeostasis. FEBS Lett. *597*, 2416–2432. https://doi.org/10.1002/1873-3468.14716.

Diwanji, N., and Bergmann, A. (2020). Basement membrane damage by ROS- and JNK-mediated Mmp2 activation drives macrophage recruitment to overgrown tissue. Nat. Commun. *11*, 3631. https://doi.org/10.1038/s41467-020-17399-8.

Dobrowolski, R., and De Robertis, E.M. (2011). Endocytic control of growth factor signalling: multivesicular bodies as signalling organelles. Nat. Rev. Mol. Cell Biol. *13*, 53–60. https://doi.org/10.1038/nrm3244.

Esteban-Collado, J., Fernàndez-Mañas, M., Fernández-Moreno, M., Maeso, I., Corominas, M., and Serras, F. (2023). ROS-mediated TNFR Wengen activation in response to apoptosis. Preprint at bioRxiv. https://doi.org/10.1101/2023.11.13.566843.

Francis, C.R., Kincross, H., and Kushner, E.J. (2022). Rab35 governs apicobasal polarity through regulation of actin dynamics during sprouting angiogenesis. Nat. Commun. *13*, 5276. https://doi.org/10.1038/s41467-022-32853-5.

Fuller, M.T., and Spradling, A.C. (2007). Male and female Drosophila germline stem cells: two versions of immortality. Science *316*, 402–404. https://doi.org/10.1126/science.1140861.

Gan, T., Fan, L., Zhao, L., Misra, M., Liu, M., Zhang, M., and Su, Y. (2021). JNK Signaling in Drosophila Aging and Longevity. Int. J. Mol. Sci. *22*, 9649. https://doi.org/10.3390/ijms22179649.

Greenspan, L.J., de Cuevas, M., Le, K.H., Viveiros, J.M., and Matunis, E.L. (2022). Activation of the EGFR/MAPK pathway drives transdifferentiation of quiescent niche cells to stem cells in the Drosophila testis niche. eLife *11*, e70810. https://doi.org/10.7554/eLife.70810.

Herrera, S.C., Sainz de la Maza, D., Grmai, L., Margolis, S., Plessel, R., Burel, M., O'Connor, M., Amoyel, M., and Bach, E.A. (2021). Proliferative stem cells maintain quiescence of their niche by secreting the Activin inhibitor Follistatin. Dev. Cell *56*, 2284–2294.e6. https://doi.org/10.1016/j.devcel.2021.07.010.

Herrera, S.C., and Bach, E.A. (2018). JNK signaling triggers spermatogonial dedifferentiation during chronic stress to maintain the germline stem cell pool in the Drosophila testis. eLife *7*, e36095. https://doi.org/10.7554/eLife.36095.

Herrera, S.C., and Bach, E.A. (2021). The Emerging Roles of JNK Signaling in Drosophila Stem Cell Homeostasis. Int. J. Mol. Sci. *22*, 5519. https://doi.org/10.3390/ijms22115519.

Hudson, A.G., Parrott, B.B., Qian, Y., and Schulz, C. (2013). A temporal signature of epidermal growth factor signaling regulates the differentiation of germline cells in testes of Drosophila melanogaster. PLoS One *8*, e70678. https://doi.org/10.1371/journal.pone.0070678.

Klinkert, K., and Echard, A. (2016). Rab35 GTPase: A Central Regulator of Phosphoinositides and F-actin in Endocytic Recycling and Beyond. Traffic *17*, 1063–1077. https://doi.org/10.1111/tra.12422.

Kouranti, I., Sachse, M., Arouche, N., Goud, B., and Echard, A. (2006). Rab35 regulates an endocytic recycling pathway essential for the terminal steps of cytokinesis. Curr. Biol. *16*, 1719–1725. https://doi.org/10.1016/j.cub.2006.07.020.

Kucinski, I., Dinan, M., Kolahgar, G., and Piddini, E. (2017). Chronic activation of JNK JAK/STAT and oxidative stress signalling causes the loser cell status. Nat. Commun. *8*, 136. https://doi.org/10.1038/s41467-017-00145-y.

La Marca, J.E., Diepstraten, S.T., Hodge, A.L., Wang, H., Hart, A.H., Richardson, H.E., and Somers, W.G. (2019). Strip and Cka negatively regulate JNK signalling during Drosophila spermatogenesis. Development *146*, dev174292. https://doi.org/10.1242/dev.174292.

Lennicke, C., and Cochemé, H.M. (2021). Redox metabolism: ROS as specific molecular regulators of cell signaling and function. Mol. Cell *81*, 3691–3707. https://doi.org/10.1016/j.molcel.2021.08.018.

Letizia, A., Espinàs, M.L., Giannios, P., and Llimargas, M. (2023). The TNFR Wengen regulates the FGF pathway by an unconventional mechanism. Nat. Commun. *14*, 5874. https://doi.org/10.1038/s41467-023-41549-3.

Loudhaief, R., Jneid, R., Christensen, C.F., Mackay, D.J., Andersen, D.S., and Colombani, J. (2023). The Drosophila tumor necrosis factor receptor, Wengen, couples energy expenditure with gut immunity. Sci. Adv. *9*, eadd4977. https://doi.org/10.1126/sciadv.add4977.

Morimoto, H., Kanastu-Shinohara, M., Ogonuki, N., Kamimura, S., Ogura, A., Yabe-Nishimura, C., Mori, Y., Morimoto, T., Watanabe, S., Otsu, K., et al. (2019). ROS amplification drives mouse spermatogonial stem cell self-renewal. Life Sci. Alliance *2*, e201900374. https://doi.org/10.26508/lsa.201900374.

Ochi, Y., Yamashita, H., Yamada, Y., Satoh, T., and Satoh, A.K. (2022). Stratum is required for both apical and basolateral transport through stable expression of Rab10 and Rab35 in Drosophila photoreceptors. Mol. Biol. Cell *33*, br17. https://doi.org/10.1091/mbc.E21-12-0596.

Palmerini, V., Monzani, S., Laurichesse, Q., Loudhaief, R., Mari, S., Cecatiello, V., Olieric, V., Pasqualato, S., Colombani, J., Andersen, D.S., and Mapelli, M. (2021). Drosophila TNFRs Grindelwald and Wengen bind Eiger with different affinities and promote distinct cellular functions. Nat. Commun. *12*, 2070. https://doi.org/10.1038/s41467-021-22080-9.

Papagiannouli, F. (2022). Endocytosis at the Crossroad of Polarity and Signaling Regulation: Learning from Drosophila melanogaster and Beyond. Int. J. Mol. Sci. *23*, 4684. https://doi.org/10.3390/ijms23094684.

Papagiannouli, F., Berry, C.W., and Fuller, M.T. (2019). The Dlg Module and Clathrin-Mediated Endocytosis Regulate EGFR Signaling and Cyst Cell-Germline Coordination in the Drosophila Testis. Stem Cell Rep. *12*, 1024–1040. https://doi.org/10.1016/j.stemcr.2019.03.008.

Patel, P.H., Pénalva, C., Kardorff, M., Roca, M., Pavlović, B., Thiel, A., Teleman, A.A., and Edgar, B.A. (2019). Damage sensing by a Nox-Ask1-MKK3-p38 signaling pathway mediates regeneration in the adult Drosophila midgut. Nat. Commun. *10*, 4365. https://doi.org/10.1038/s41467-019-12336-w.

Sakauchi, C., Wakatsuki, H., Ichijo, H., and Hattori, K. (2017). Pleiotropic properties of ASK1. Biochim. Biophys. Acta Gen.

Subj. *1861*, 3030–3038. https://doi.org/10.1016/j.bbagen.2016.09.028.

Santabarbara-Ruiz, P., Esteban-Collado, J., Perez, L., Viola, G., Abril, J.F., Milan, M., Corominas, M., and Serras, F. (2019). Ask1 and Akt act synergistically to promote ROS-dependent regeneration in Drosophila. PLoS Genet. *15*, e1007926. https://doi.org/10.1371/journal.pgen.1007926.

Santabarbara-Ruiz, P., Lopez-Santillan, M., Martinez-Rodriguez, I., Binagui-Casas, A., Perez, L., Milan, M., Corominas, M., and Serras, F. (2015). ROS-Induced JNK and p38 Signaling Is Required for Unpaired Cytokine Activation during Drosophila Regeneration. PLoS Genet. *11*, e1005595. https://doi.org/10.1371/journal.pgen.1005595.

Sarkar, A., Parikh, N., Hearn, S.A., Fuller, M.T., Tazuke, S.I., and Schulz, C. (2007). Antagonistic roles of Rac and Rho in organizing the germ cell microenvironment. Curr. Biol. *17*, 1253–1258. https://doi.org/10.1016/j.cub.2007.06.048.

Schieber, M., and Chandel, N.S. (2014). ROS function in redox signaling and oxidative stress. Curr. Biol. *24*, R453–R462. https://doi.org/10.1016/j.cub.2014.03.034.

Senos Demarco, R., and Jones, D.L. (2019). Mitochondrial fission regulates germ cell differentiation by suppressing ROS-mediated activation of Epidermal Growth Factor Signaling in the Drosophila larval testis. Sci. Rep. *9*, 19695. https://doi.org/10.1038/s41598-019-55728-0.

Senos Demarco, R., and Jones, D.L. (2020). EGFR signaling promotes basal autophagy for lipid homeostasis and somatic stem cell maintenance in the Drosophila testis. Autophagy *16*, 1145–1147. https://doi.org/10.1080/15548627.2020.1739450.

Senos Demarco, R., Uyemura, B.S., and Jones, D.L. (2020). EGFR Signaling Stimulates Autophagy to Regulate Stem Cell Maintenance and Lipid Homeostasis in the Drosophila Testis. Cell Rep. *30*, 1101–1116.e1105. https://doi.org/10.1016/j.celrep.2019.12.086.

Serizier, S.B., Peterson, J.S., and McCall, K. (2022). Non-autonomous cell death induced by the Draper phagocytosis receptor requires signaling through the JNK and SRC pathways. J. Cell Sci. *135*, jcs250134. https://doi.org/10.1242/jcs.250134.

Serras, F. (2022). The sooner, the better: ROS, kinases and nutrients at the onset of the damage response in Drosophila. Front. Cell Dev. Biol. *10*, 1047823. https://doi.org/10.3389/fcell.2022.1047823.

Shim, J., Lee, S.M., Lee, M.S., Yoon, J., Kweon, H.S., and Kim, Y.J. (2010). Rab35 mediates transport of Cdc42 and Rac1 to the plasma membrane during phagocytosis. Mol. Cell Biol. *30*, 1421–1433. https://doi.org/10.1128/MCB.01463-09.

Shinzawa, N., Nelson, B., Aonuma, H., Okado, K., Fukumoto, S., Miura, M., and Kanuka, H. (2009). p38 MAPK-dependent phagocytic encapsulation confers infection tolerance in Drosophila. Cell Host Microbe *6*, 244–252. https://doi.org/10.1016/j.chom.2009.07.010.

Sinenko, S.A., Starkova, T.Y., Kuzmin, A.A., and Tomilin, A.N. (2021). Physiological Signaling Functions of Reactive Oxygen Species in Stem Cells: From Flies to Man. Front. Cell Dev. Biol. *9*, 714370. https://doi.org/10.3389/fcell.2021.714370.

Stephens, R., Lim, K., Portela, M., Kvansakul, M., Humbert, P.O., and Richardson, H.E. (2018). The Scribble Cell Polarity Module in the Regulation of Cell Signaling in Tissue Development and Tumorigenesis. J. Mol. Biol. *430*, 3585–3612. https://doi.org/10.1016/j.jmb.2018.01.011.

Sun, X., Lu, L., Wang, K., Song, L., Jiao, J., Wu, Y., Wang, X., Song, Y., and Zhan, L. (2023). Scribble deficiency mediates colon inflammation by inhibiting autophagy-dependent oxidative stress elimination. Sci. Rep. *13*, 18327. https://doi.org/10.1038/s41598-023-45176-2.

Takeda, K., Noguchi, T., Naguro, I., and Ichijo, H. (2008). Apoptosis signal-regulating kinase 1 in stress and immune response. Annu. Rev. Pharmacol. Toxicol. *48*, 199–225. https://doi.org/10.1146/annurev.pharmtox.48.113006.094606.

Tan, S.W.S., Lee, Q.Y., Wong, B.S.E., Cai, Y., and Baeg, G.H. (2017). Redox Homeostasis Plays Important Roles in the Maintenance of the Drosophila Testis Germline Stem Cells. Stem Cell Rep. *9*, 342–354. https://doi.org/10.1016/j.stemcr.2017.05.034.

Tang, Y., Geng, Q., Chen, D., Zhao, S., Liu, X., and Wang, Z. (2017). Germline Proliferation Is Regulated by Somatic Endocytic Genes via JNK and BMP Signaling in Drosophila. Genetics *206*, 189–197. https://doi.org/10.1534/genetics.116.196535.

Uhlirova, M., and Bohmann, D. (2006). JNK- and Fos-regulated Mmp1 expression cooperates with Ras to induce invasive tumors in Drosophila. EMBO J. *25*, 5294–5304. https://doi.org/10.1038/sj.emboj.7601401.

Wagner, E.F., and Nebreda, A.R. (2009). Signal integration by JNK and p38 MAPK pathways in cancer development. Nat. Rev. Cancer *9*, 537–549. https://doi.org/10.1038/nrc2694.

Welz, T., Wellbourne-Wood, J., and Kerkhoff, E. (2014). Orchestration of cell surface proteins by Rab11. Trends Cell Biol. *24*, 407–415. https://doi.org/10.1016/j.tcb.2014.02.004.

Xi, G., Lamba, S.A., Mysh, M., and Poulton, J.S. (2024). Oxidative Stress Contributes to Slit Diaphragm Defects Caused by Disruption of Endocytosis. Kidney Int. Rep. *9*, 451–463. https://doi.org/10.1016/j.ekir.2023.11.018.

Zohar-Fux, M., Ben-Hamo-Arad, A., Arad, T., Volin, M., Shklyar, B., Hakim-Mishnaevski, K., Porat-Kuperstein, L., Kurant, E., and Toledano, H. (2022). The phagocytic cyst cells in Drosophila testis eliminate germ cell progenitors via phagoptosis. Sci. Adv. *8*, eabm4937. https://doi.org/10.1126/sciadv.abm4937.

Zoller, R., and Schulz, C. (2012). The Drosophila cyst stem cell lineage: Partners behind the scenes? Spermatogenesis *2*, 145–157. https://doi.org/10.4161/spmg.21380.

Stem Cell Reports, Volume *20*

Supplemental Information

Interplay of EGFR, JNK, and ROS signaling in soma-germline communication in the *Drosophila* testis

Maria Alvarez and Fani Papagiannouli

SUPPLEMENTAL METHODS

Fly stocks and husbandry

The following stocks were from the Bloomington Stock Center (BL) Indiana: *UAS-scrib-RNAi*$^{TRiP.HMS01490}$, *UAS-AP2α (adaptin)-RNAi*$^{TRiP.HMS00653}$, *UAS-shi-RNAi*$^{TRiP.JF03133}$, *UAS-EGFR*^{CA} (BL9533), *UAS-mCD8-GFP* (BL5139), *Pin/CyO; UAS-mCD8-GFP* (BL5130), *αtub84B-GAL80*^{ts}*/TM2* (BL7017), *αtub-GAL80*^{ts}*; TM2/TM6B,Tb* (BL7019), *UAS-Basket-RNAi*$^{TRiP.JF01275}$ (BL31323), *UAS-Rab35-RNAi*$^{TRiP.JF02978}$ (BL28342), *UAS-Ask1-RNAi*$^{TRiP.HMS300464}$ (BL32464), *UAS-wengen-RNAi*$^{TRiP.HMC03962}$ (BL55275), *UAS-Nox-RNAi*$^{TRiP.HMS00691}$ (BL32902), *UAS-Duox-RNAi*$^{TRiP.HMS00629}$, (BL32846). The following stocks used in this study were from the Vienna *Drosophila* RNAi Center (VDRC) Austria: *UAS-dlg-RNAi*^{v41134}, *UAS-dlg-RNAi*^{v41136}*/TM3*, *UAS-lgl-RNAi*^{v109604}, *UAS-lgl-RNAi*^{v51247}, *UAS-Chc-RNAi*^{v103383}, *UAS-Chc-RNAi*^{v32666}, *UAS-p38a-RNAi*^{v342386}, *UAS-grindewald-RNAi*^{v104538}, *UAS-lic-RNAi*^{v106822}, *Rab35-FlyFos (v318284), wgn-FlyFos (v318644)*. All RNAi stocks used in this study have been effective in knocking down the corresponding genes in previous studies (Brantley and Fuller, 2019; Fujisawa et al., 2020; Papagiannouli et al., 2019; Patel et al., 2019).

The *c587-GAL4* was obtained from Margaret Fuller, JNK reporter *TRE-GFP (attP16)* thereafter mentioned as *puc::TRE-GFP* (Chatterjee and Bohmann, 2012) was obtained from David Bilder. Other fly stocks used in this study are described in FlyBase (www.flybase.org). Genomics EGFR-GFP transgenic fly lines were a gift of Bassem Hassan (Zschatzsch et al., 2014). All *UAS-gene*^{RNAi} stocks are referred to in the text as *gene*^{RNAi} for simplicity reasons. Knockdowns were performed using the *UAS-GAL4* system (Brand and Perrimon, 1993) by combining the *UAS-RNAi* fly lines with the cell-type specific *c587-GAL4* driver (Kai and Spradling, 2004) and *αtub-Gal80*^{ts} (Lee and Luo, 1999).

For the phenotypic analysis in adult *Drosophila* testes: *c587*-GAL4; *αtub-Gal80*^{ts}*; UAS-mCD8-GFP* or *c587*-GAL4*; αtub-Gal80*^{ts} flies were crossed to *UAS-gene*^{RNAi} flies. Crosses were raised at 18°C until adult flies hatched. Then adult males 1-3 days old with the correct genotype (along with few females in order to mate) were shifted at 30°C for 4 or 7 days depending on the experimental needs and the phenotypes were analysed. To control for possible effects of multiple *UAS* constructs limiting the effectiveness of the *GAL4* driver, control flies carried the same number of *UAS* transgenes using a *UAS-mCD8-GFP* (we could not use a *UAS-GFP-RNAi* line, as this would interfere with the *puc::TRE-GFP*).

Immunofluorescence staining and microscopy

The monoclonal antibodies used in this study: anti-Vasa (1/10; rat), anti-Mmp1 antibodies 3B8D12, 5H7B11, 3A6B4 (each used in 1/10 dilution) were obtained from the Developmental Studies Hybridoma Bank developed under the auspices of the NICHD and maintained by The University of

Iowa, Department of Biological Sciences, Iowa City, IA 52242. Polyclonal chicken anti-GFP (13970; 1/10,000) was from Abcam, polyclonal rabbit Phospho-p38 MAPK (Thr180/Tyr182) Antibody (9211; 1/200) from Cell Signaling. Filamentous (F-actin) was stained with Alexa Fluor phalloidin 546 (1/300, Thermo Fischer Scientific) and DNA with DAPI (Thermo Fischer Scientific) or in DAPI containing mounting medium. Following secondary antibodies were used: donkey anti-mouse Alexa Fluor-546 and Alexa Fluor-647, donkey anti-rat Alexa Fluor-647, donkey anti-rabbit Alexa Fluor-488 and donkey anti-chicken Alexa Fluor-488 from Thermo Fischer Scientific (1/500).

For whole mount testes immunostaining, testes were dissected in PBS, fixed for 20min in 8% formaldehyde (FA) and rinsed twice in 1% PBX (1% Triton-100x in PBS). Testes were blocked in 5% Bovine Serum Albumin in 1% PBX for 1h. Testes were incubated with primary antibodies over-night at 4°C and the following day with the secondary antibodies for 2h at room temperature (RT) in the dark (Papagiannouli *et al.*, 2019). For testes immunostaining in the presence of GFP, 1% PBT (1% Tween-20 in PBS) was used instead of 1% PBX in all steps. For the TUNEL assay, tissue was processed as described above except that after fixation, the protocol from In Situ Cell Death Detection Kit (TMR Red, Sigma/Roche) was followed (Papagiannouli *et al.*, 2019). For the Mmp1 staining, DHSB antibodies 3B8D12, 5H7B11, 3A6B4 where mixed in equal amounts (1:1:1 ratio) and the mix was used in a 3/10 dilution. Testes were blocked O/N and incubated in primary antibody mix for 72h. Staining with the polyclonal rabbit Phospho-p38 MAPK antibody gave unspecific sticky staining in spermatocytes, in which case stainings were optimised by preabsorbing the antibody for 2 days with *c587>UAS-mCD8-GFP* testes, before use. Stainings with anti-GFP antibody against Wgn-GFP and EGFR-GFP transgenes were extremely weak, thus, to combat this we incubated the testes for 2 days in the blocking solution to prevent unspecific sticky staining in spermatocytes, and we raised the concentration of anti-GFP to 1/600 dilution. Previous studies on nerve axon branches, also observed too low expression levels of the genomic EGFR-GFP transgene to allow analysis at sub-cellular (Zschatzsch *et al.*, 2014). Testes were mounted in ProLong® Gold Antifade with DAPI (P36931; Thermo Fischer Scientific).

Treatment of flies with antioxidant Vitamin C was performed by feeding male flies with food containing 100mM Vitamin C (L-ascorbic acid; Merck – A4403) for the two days before dissection.

Confocal images were obtained using a Zeiss LSM880 with Airy scan (1024x1024px, 225μm image frame) (University of Greenwich at Medway Campus). Pictures were finally processed with Adobe Photoshop 7.0.

Quantification of fluorescent images and Statistical analysis

Quantifications were done using FiJi/ImageJ by measuring “Corrected Total Cell Fluorescence” (CTCF) [CTCF = Integrated Density - (Area of elected cell X Mean fluorescence of background

readings)] using Excel. Statistical analysis was performed in GraphPad/Prism. The control (in most cases *c587-GAL4* or *c587>UAS-mCD8* testes reflecting physiological levels) was compared to each individual sample using the non-normal, non-parametric two-sampled Mann–Whitney test (also known as Wilcoxon test). Comparisons with a P-value ≥0.05 were marked as 'ns' (not significant); *p<0.05; **p<0.01; ***p<0.001; ****p<0.0001.

ROS staining protocol

A Dihydroethidium (DHE) (D11347, 10x1mg, Invitrogen™) 30mM stock solution was prepared using anhydrous DMSO (Sigma-Aldrich, cat. no. 276855) and kept in small aliquots at -20°C until used. Testes were dissected in DMEM or Schneiders medium (both worked equally well in our hands). 30mM stock was diluted to a 30μM (1/1000) working concentration with DMEM medium and mixed well with vortex, giving rise to a pink solution. Testes were incubated in this mix for 7min in the dark, on an orbital shaker at RT. DHE solution was removed and traces were removed by incubating testes 3 times on DMEM medium for 5 min. Testes were fixed for 4min in 8% formaldehyde, rinsed once with PBS, mounted in ProLong® Gold Antifade with DAPI and imaged immediately with confocal microscope.

REFERENCES

Brand, A.H., and Perrimon, N. (1993). Targeted gene expression as a means of altering cell fates and generating dominant phenotypes. Development *118*, 401-415.

Brantley, S.E., and Fuller, M.T. (2019). Somatic support cells regulate germ cell survival through the Baz/aPKC/Par6 complex. Development *146*. 10.1242/dev.169342.

Chatterjee, N., and Bohmann, D. (2012). A versatile PhiC31 based reporter system for measuring AP-1 and Nrf2 signaling in Drosophila and in tissue culture. PLoS One *7*, e34063. 10.1371/journal.pone.0034063.

Fujisawa, Y., Shinoda, N., Chihara, T., and Miura, M. (2020). ROS Regulate Caspase-Dependent Cell Delamination without Apoptosis in the Drosophila Pupal Notum. iScience *23*, 101413. 10.1016/j.isci.2020.101413.

Kai, T., and Spradling, A. (2004). Differentiating germ cells can revert into functional stem cells in Drosophila melanogaster ovaries. Nature *428*, 564-569. 10.1038/nature02436.

Lee, T., and Luo, L. (1999). Mosaic analysis with a repressible cell marker for studies of gene function in neuronal morphogenesis. Neuron *22*, 451-461.

Papagiannouli, F., Berry, C.W., and Fuller, M.T. (2019). The Dlg Module and Clathrin-Mediated Endocytosis Regulate EGFR Signaling and Cyst Cell-Germline Coordination in the Drosophila Testis. Stem cell reports *12*, 1024-1040. 10.1016/j.stemcr.2019.03.008.

Patel, P.H., Penalva, C., Kardorff, M., Roca, M., Pavlovic, B., Thiel, A., Teleman, A.A., and Edgar, B.A. (2019). Damage sensing by a Nox-Ask1-MKK3-p38 signaling pathway mediates regeneration in the adult Drosophila midgut. Nature communications *10*, 4365. 10.1038/s41467-019-12336-w.

Zschatzsch, M., Oliva, C., Langen, M., De Geest, N., Ozel, M.N., Williamson, W.R., Lemon, W.C., Soldano, A., Munck, S., Hiesinger, P.R., et al. (2014). Regulation of branching dynamics by axon-intrinsic asymmetries in Tyrosine Kinase Receptor signaling. eLife *3*, e01699. 10.7554/eLife.01699.

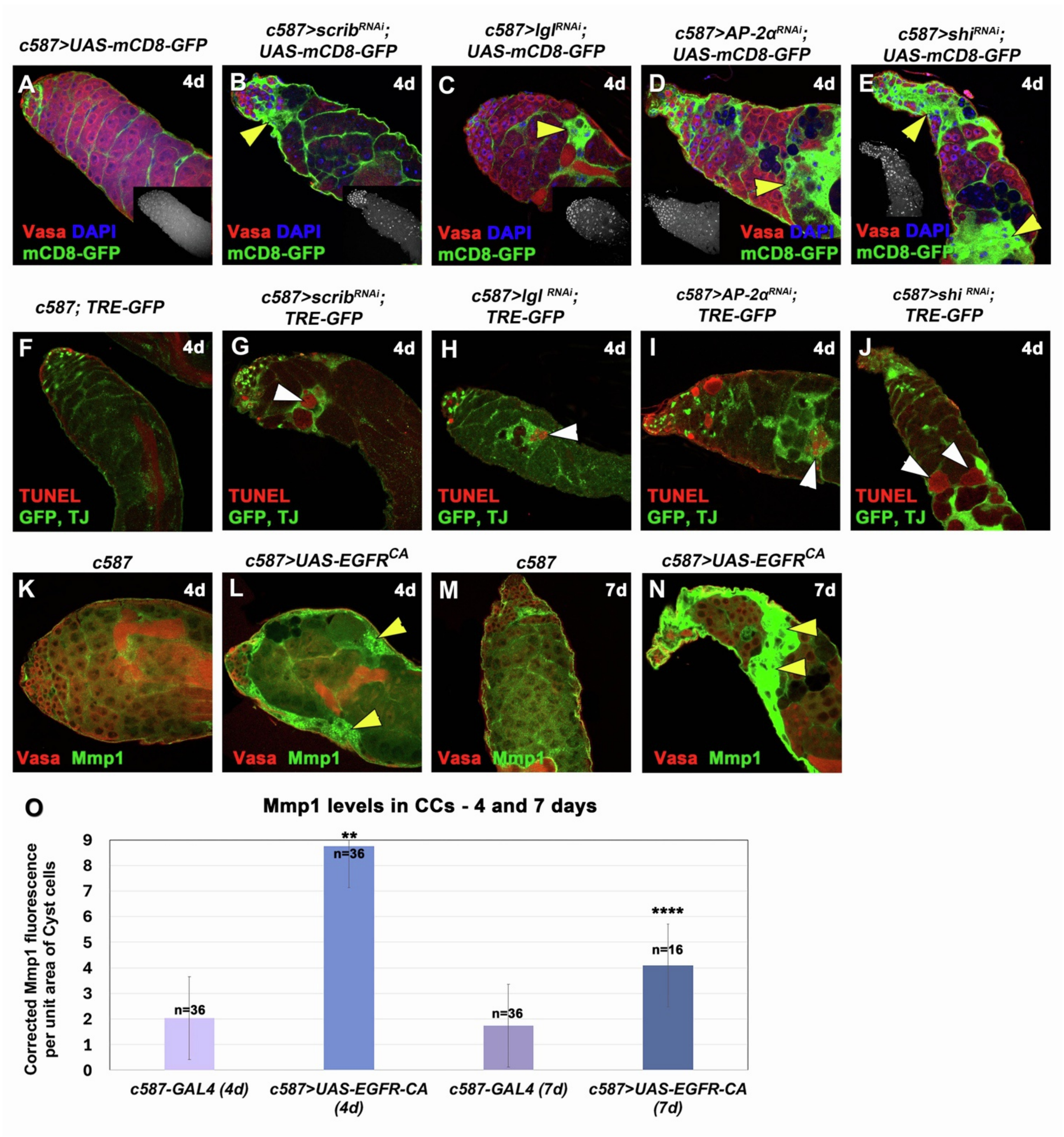

Figure S1: Overactivation of EGFR, also via knockdown of *scrib, lgl, AP-2α* or *shi* function, in cyst cells leads to activation of JNK signaling in cyst cells and apoptosis in the neighbouring germline, related to Figure 1**.** Adult testes of the indicated genotypes in the *Gal80^{ts}* background: **(A-E)** *mCD8-GFP* (green; CCs), Vasa (red; germline), DAPI (blue; nuclei). Yellow arrowheads: mCD8+ CC regions. **(F-J)** TUNEL (red; apoptotic double-strand breaks), AP-1 responsive TRE elements corresponding to JNK reporter *puc* expression levels (*TRE-GFP*) and TJ (early CCs) (green). White arrowheads: dying germ cells (spermatogonia and spermatocytes) surrounded by CCs with upregulated JNK levels. **(K-N)** levels of the Mmp1 protein in CCs (green); Vasa (red; germline). Yellow arrowheads: Mmp1 in CCs. *UAS* activated at 30°C for 4 or 7 days (d). **(O)** Quantification of corrected fluorescent Mmp1 levels in CCs (4- and 7-days activation). Each individual sample was

compared to control (error bars: standard error; ns: not significant; *$p<0.05$; **$p<0.01$; ***$p<0.001$; ****$p<0.0001$). Numbers (n) in each column represent sample size. Testes oriented with anterior at left. Image frames (A-N): 225μm

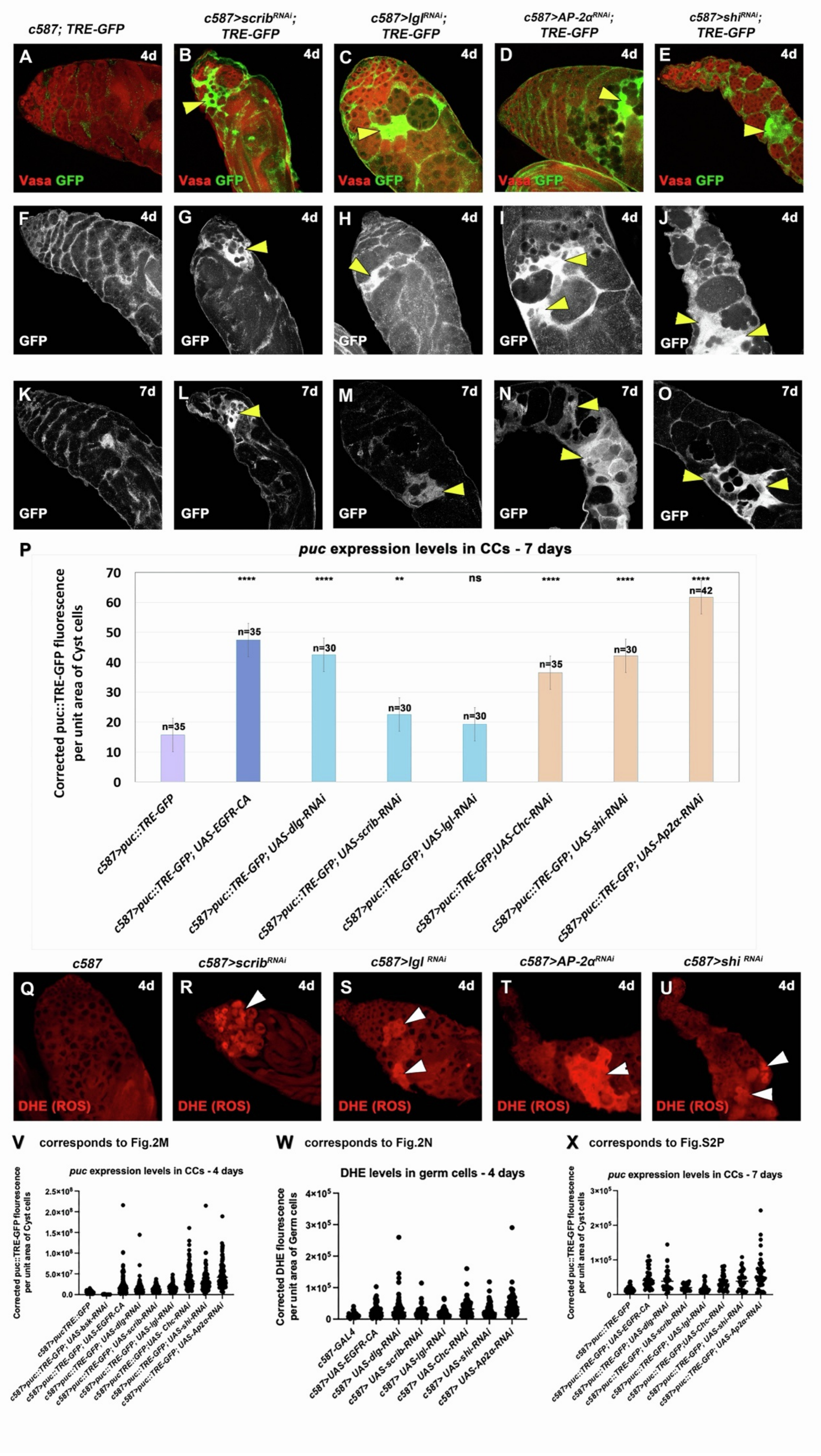

c587; TRE-GFP
c587>scrib^RNAi; TRE-GFP
c587>lgl^RNAi; TRE-GFP
c587>AP-2α^RNAi; TRE-GFP
c587>shi^RNAi; TRE-GFP
A B C D E
4d
Vasa GFP
F G H I J
GFP
K L M N O
7d
P
puc expression levels in CCs - 7 days
Corrected puc::TRE-GFP fluorescence per unit area of Cyst cells
70
60
50
40
30
20
10
0
**** **** ** ns **** **** ****
n=35 n=35 n=30 n=30 n=30 n=35 n=30 n=42
c587>puc::TRE-GFP
c587>puc::TRE-GFP; UAS-EGFR-CA
c587>puc::TRE-GFP; UAS-dlg-RNAi
c587>puc::TRE-GFP; UAS-scrib-RNAi
c587>puc::TRE-GFP; UAS-lgl-RNAi
c587>puc::TRE-GFP;UAS-Chc-RNAi
c587>puc::TRE-GFP; UAS-shi-RNAi
c587>puc::TRE-GFP; UAS-Ap2α-RNAi
c587
c587>scrib^RNAi
c587>lgl ^RNAi
c587>AP-2α^RNAi
c587>shi ^RNAi
Q R S T U
DHE (ROS)
V corresponds to Fig.2M
puc expression levels in CCs - 4 days
W corresponds to Fig.2N
DHE levels in germ cells - 4 days
Corrected DHE flourescence per unit area of Germ cells
X corresponds to Fig.S2P
puc expression levels in CCs - 7 days
Corrected puc::TRE-GFP flourescence per unit area of Cyst cells

Figure S2: Overexpression of EGFR or knockdown of *scrib, lgl* or *AP-2α, shi* function, in cyst cells leads to increased levels of JNK signaling in the cyst cells and ROS in the germline, related to Figure 2**.** Adult testes of the indicated genotypes in the *Gal80^{ts}* background. **(A-O)** *TRE-GFP* reflects expression levels of JNK reporter *puc* in CCs for 4 days (A-J) and 7 days (K-O) activation. (A-E) *TRE-GFP* (green; CCs); Vasa (red; germline). (F-O) show the *puc::TRE-GFP* levels only (white) in directly comparable raw images. Yellow arrowheads: regions of *puc::TRE* overactivation in CCs. **(P)** Quantification of corrected fluorescent *puc::TRE-GFP* levels in CCs (7 days activation). Each individual sample was compared to control (error bars: standard error; ns: not significant; *p<0.05; **p<0.01; ***p<0.001; ****p<0.0001). Numbers (n) in each column represent sample size. **(Q-U)** DHE (red; germ cells) reflects ROS activation in the germ cells. White arrowheads: representative areas of ROS activation in the germline. *UAS* activated at 30°C for 4 days (4d). **(V-X)** Dotted plots for quantifications of Figures 2M, 2N and the S2P (above). Testes oriented with anterior at left. Image frames (A-O, Q-U): 225μm

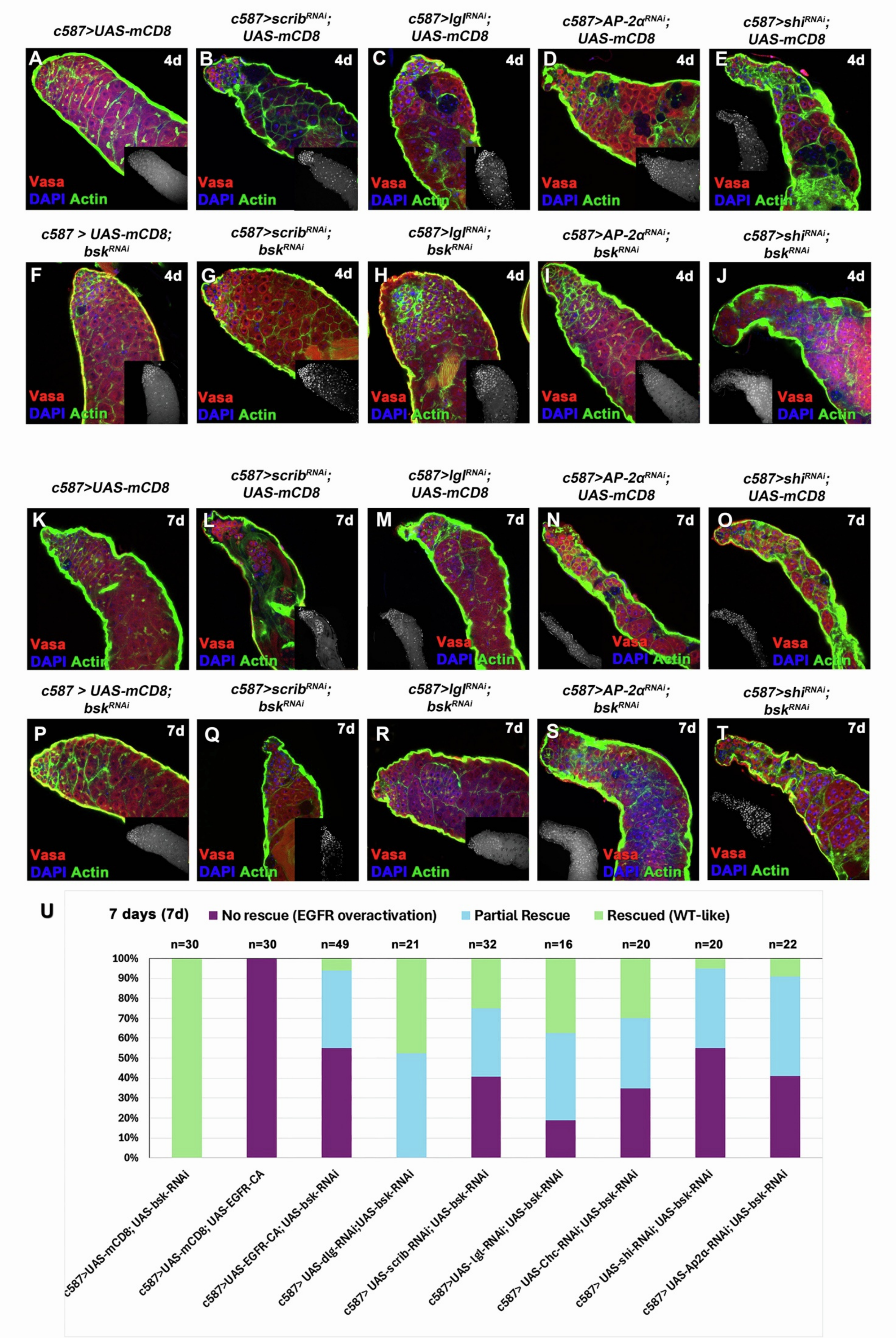
c587>UAS-mCD8
c587>scrib^RNAi; UAS-mCD8
c587>lgl^RNAi; UAS-mCD8
c587>AP-2α^RNAi; UAS-mCD8
c587>shi^RNAi; UAS-mCD8
c587 > UAS-mCD8; bsk^RNAi
c587>scrib^RNAi; bsk^RNAi
c587>lgl^RNAi; bsk^RNAi
c587>AP-2α^RNAi; bsk^RNAi
c587>shi^RNAi; bsk^RNAi
4d
7d
Vasa
DAPI Actin
U
7 days (7d)
No rescue (EGFR overactivation)
Partial Rescue
Rescued (WT-like)
n=30
n=30
n=49
n=21
n=32
n=16
n=20
n=20
n=22
100%
90%
80%
70%
60%
50%
40%
30%
20%
10%
0%
c587>UAS-mCD8; UAS-bsk-RNAi
c587>UAS-mCD8; UAS-EGFR-CA
c587>UAS-EGFR-CA; UAS-bsk-RNAi
c587> UAS-dlg-RNAi;UAS-bsk-RNAi
c587> UAS-scrib-RNAi; UAS-bsk-RNAi
c587>UAS- lgl-RNAi; UAS-bsk-RNAi
c587> UAS-Chc-RNAi; UAS-bsk-RNAi
c587> UAS-shi-RNAi; UAS-bsk-RNAi
c587> UAS-Ap2α-RNAi; UAS-bsk-RNAi

Figure S3: Knocking down the JUN kinase *bsk* in cyst cells, can partially rescue the germ cell death phenotype observed upon EGFR overactivation, related to Figure 3**. (A-T)** Adult testes of the indicated genotypes in the *Gal80^{ts}* background: Vasa (red; germline), DAPI (blue; nuclei) and Actin stained with phalloidin (here shown in the green channel; hub, CySCs, CCs and germline fusome) also in flies containing the *mCD8-GFP* transgene (since the GFP is not shown here). *UAS* activated at 30°C for 4 (A-J) and 7 (K-T) days (d). **(U)** Quantifications of the different phenotypic classes accompanying each genotype, organized in order of phenotypic strength (4- and 7- days activation). Numbers (n) in each column represent sample size. Testes oriented with anterior at left. Image frames (A-T): 225μm.

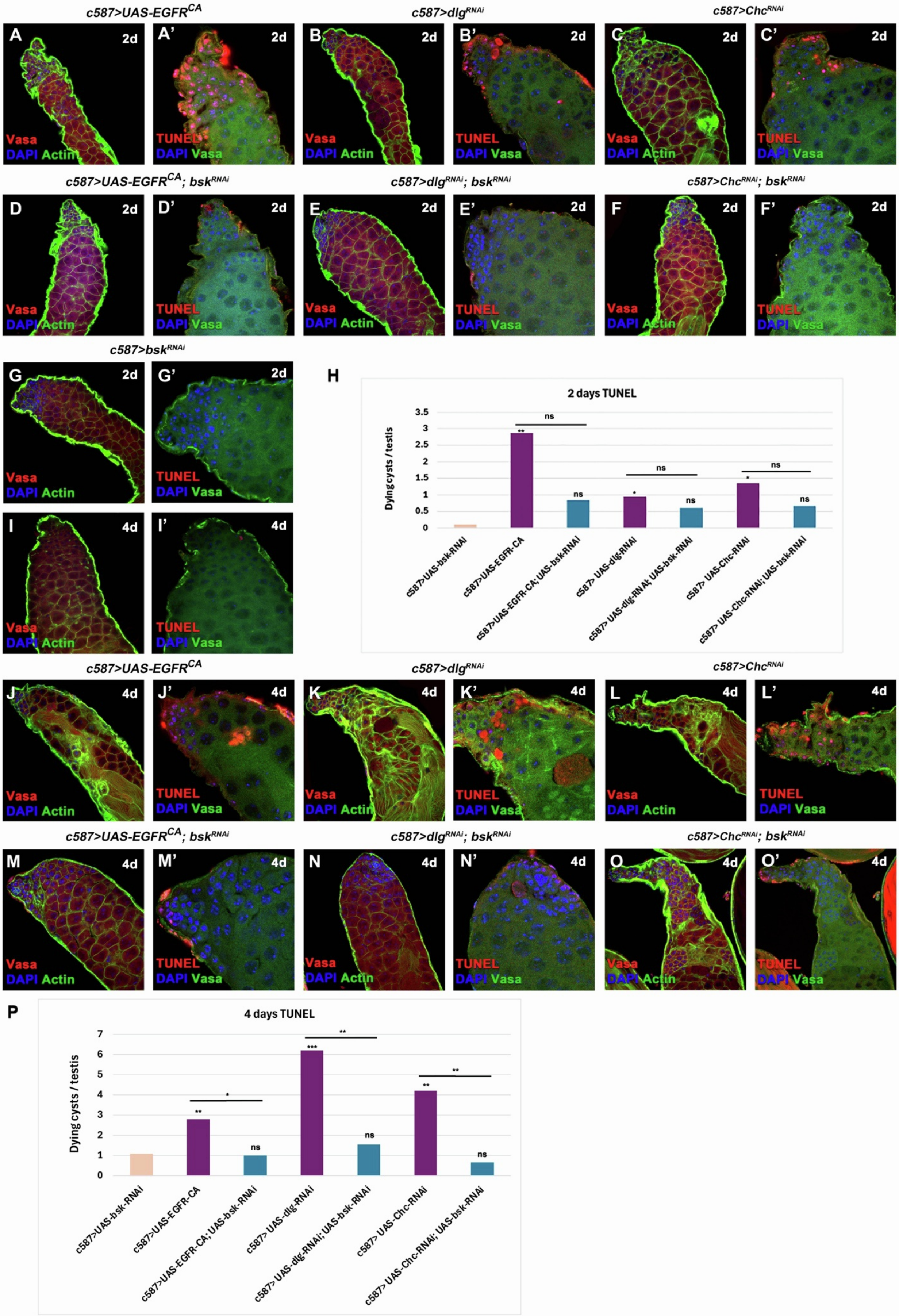
c587>UAS-EGFR^CA
c587>dlg^RNAi
c587>Chc^RNAi
c587>UAS-EGFR^CA; bsk^RNAi
c587>dlg^RNAi; bsk^RNAi
c587>Chc^RNAi; bsk^RNAi
c587>bsk^RNAi
Vasa
DAPI Actin
TUNEL
DAPI Vasa
2d
4d
H
2 days TUNEL
Dying cysts / testis
ns
**
*
c587>UAS-bsk-RNAi
c587>UAS-EGFR-CA
c587>UAS-EGFR-CA; UAS-bsk-RNAi
c587> UAS-dlg-RNAi
c587> UAS-dlg-RNAi; UAS-bsk-RNAi
c587> UAS-Chc-RNAi
c587> UAS-Chc-RNAi; UAS-bsk-RNAi
P
4 days TUNEL
***

Figure S4: Lowering the levels of the JUN kinase *basket* in cyst cells showed significant reduction in germ cell death (GCD), related to Figure 4**.** Adult testes of the indicated genotypes in the *Gal80*^{ts} background. *UAS* activation was performed for 2 days in (A-G') and 4 days in (I-O')**. (A-G** and **I-O)** Vasa (red; germline), DAPI (blue; nuclei) and Actin stained with phalloidin (green; hub, CySCs, CCs and germline fusome) (image frames 225µm) show the overall testis structure in EGFR overactivation (A-C, J-L) vs *bsk*-RNAi rescue context (D-F, M-O) and control (G, I). Image frames 225µm. **(A'-G'** and **I'-O')** show higher magnification of (A-G and I-O) at 112.5µm with TUNEL (red; apoptosis), Vasa (here green; germline), DAPI (blue; nuclei) for a better visualization of the dying germ cell cysts. **(H)** and **(P)** Quantifications of dying germline cysts per testes accompanying each genotype after 2- and 4- days of *UAS* activation, respectively. Each genotype was individually compared to control *c587>UAS-bsk-RNAi*, while EGFR overactivation phenotypes (columns 2, 4, 6) were compared to the corresponding bsk-mediated rescues (columns 3, 5, 7) (error bars: standard error; ns: not significant; *p<0.05; **p<0.01). Testes oriented with anterior at left.

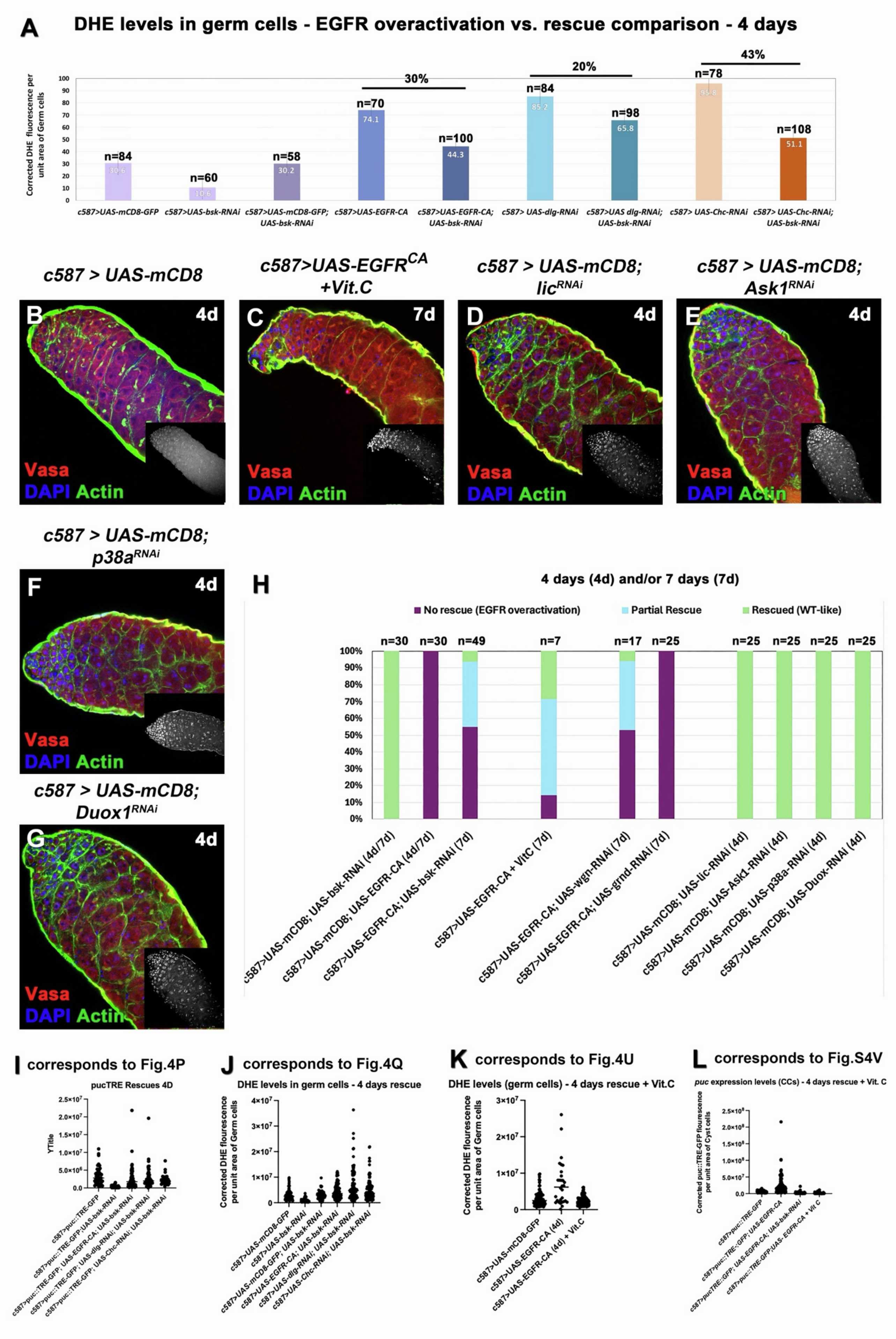

A
DHE levels in germ cells - EGFR overactivation vs. rescue comparison - 4 days
Corrected DHE fluorescence per unit area of Germ cells
30%
20%
43%
n=84
n=60
n=58
n=70
n=100
n=84
n=98
n=78
n=108
c587>UAS-mCD8-GFP
c587>UAS-bsk-RNAi
c587>UAS-mCD8-GFP; UAS-bsk-RNAi
c587>UAS-EGFR-CA
c587>UAS-EGFR-CA; UAS-bsk-RNAi
c587> UAS-dlg-RNAi
c587>UAS dlg-RNAi; UAS-bsk-RNAi
c587> UAS-Chc-RNAi
c587> UAS-Chc-RNAi; UAS-bsk-RNAi
c587 > UAS-mCD8
c587>UAS-EGFR^{CA} +Vit.C
c587 > UAS-mCD8; lic^{RNAi}
c587 > UAS-mCD8; Ask1^{RNAi}
B 4d
C 7d
D 4d
E 4d
Vasa
DAPI Actin
c587 > UAS-mCD8; p38a^{RNAi}
F 4d
c587 > UAS-mCD8; Duox1^{RNAi}
G 4d
H
4 days (4d) and/or 7 days (7d)
No rescue (EGFR overactivation)
Partial Rescue
Rescued (WT-like)
n=30 n=30 n=49 n=7 n=17 n=25 n=25 n=25 n=25 n=25
c587>UAS-mCD8; UAS-bsk-RNAi (4d/7d)
c587>UAS-mCD8; UAS-EGFR-CA (4d/7d)
c587>UAS-EGFR-CA; UAS-bsk-RNAi (7d)
c587>UAS-EGFR-CA + VitC (7d)
c587>UAS-EGFR-CA; UAS-wgn-RNAi (7d)
c587>UAS-EGFR-CA; UAS-grnd-RNAi (7d)
c587>UAS-mCD8; UAS-lic-RNAi (4d)
c587>UAS-mCD8; UAS-Ask1-RNAi (4d)
c587>UAS-mCD8; UAS-p38a-RNAi (4d)
c587>UAS-mCD8; UAS-Duox-RNAi (4d)
I corresponds to Fig.4P
pucTRE Rescues 4D
J corresponds to Fig.4Q
DHE levels in germ cells - 4 days rescue
K corresponds to Fig.4U
DHE levels (germ cells) - 4 days rescue + Vit.C
L corresponds to Fig.S4V
puc expression levels (CCs) - 4 days rescue + Vit. C

Figure S5: (A) Double knockdown of the JUN kinase *bsk* with *scrib, lgl, Ap-2α* or *shi* in cyst cells, lowers ROS levels in the germline, related to Figure 5**.** Combined quantifications of ROS levels from Fig.2N and Fig.4Q, comparing DHE levels in “EGFR overactivation” vs *bsk*-rescue backgrounds. Numbers inside the columns represent DHE levels in “EGFR overactivation” genotypes vs. rescues and % reflects this difference for each pair. Numbers (n) above each column represent sample size. As original data in Fig.2N and Fig.4Q, were obtained with a different laser, they were normalised against the *c587* controls to allow the comparison. **(B-G) Treatment with Vitamin C and knockdown of p38 pathway components in cyst cells.** Adult testes of the indicated genotypes in the $Gal80^{ts}$ background. Vasa (red; germline), DAPI (blue; nuclei) and Actin stained with phalloidin (here shown in the green channel; hub, CySCs, CCs and germline fusome) also in flies containing the *mCD8-GFP* transgene (since the GFP is not shown here). *UAS* activated at 30°C for 4 and 7 days (d). **(H)** Quantifications of the different phenotypic classes accompanying each genotype, organized in order of phenotypic strength (4- or 7- days activation) **(Matches Figure 5J).** Each individual sample was compared to control (error bars: standard error; ns: not significant; *p<0.05; **p<0.01; ***p<0.001; ****p<0.0001). Numbers (n) in each column represent sample size. **(I-L)** Dotted plots for quantifications of Figures 4P, 4Q, 4U and the S4V (above). Testes oriented with anterior at left. Image frames (A-G, I-L): 225μm

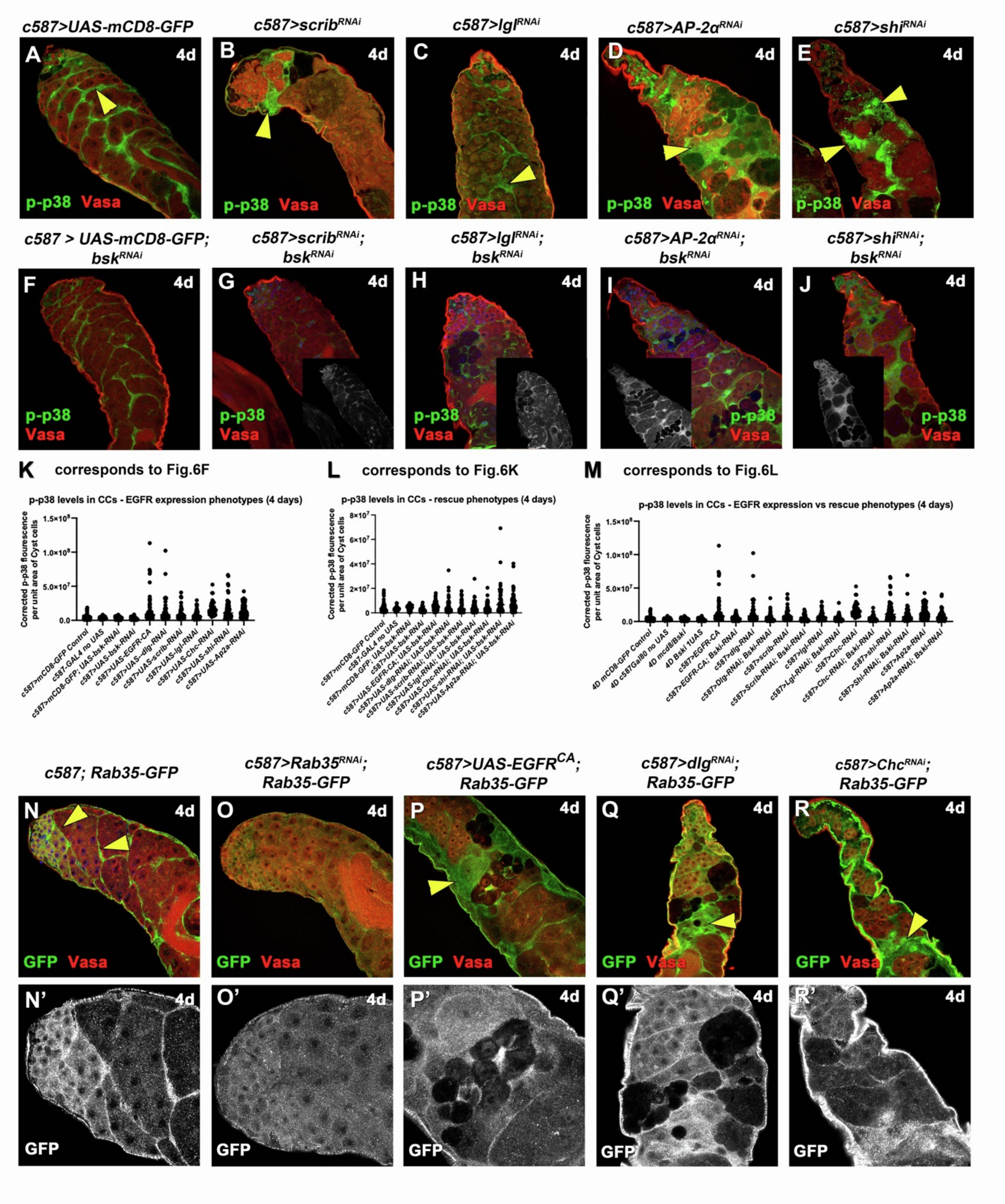

Figure S6: (A-M) Levels of phosphorylated MAPK p38 in EGFR overactivation and rescue phenotypes after knocking down the JUN kinase *basket* in cyst cells, related to Figure 6**.** Adult testes of the indicated genotypes in the *Gal80^{ts}* background: Vasa (red; germline), DAPI (blue; nuclei) and phosphorylated p38 (p-p38) (green; CCs). Yellow arrowheads point at CCs with high levels of p-p38. Small inset pictures show the p-p38 staining only. **(K-M)** Dotted plots for quantifications of Figures 6F, 6K and 6L.

(N-R') Localization of the Rab35 GTPase in cyst cells and spermatogonia, related to Figure 7**.** Adult testes of the indicated genotypes in the *Gal80^{ts}* background. **(N-R)** Vasa (red; germline), Rab35-GFP (green; CCs and early spermatogonia). Yellow arrowheads point at Rab35 staining in the CCs. **(N'-R')** show the Rab35-GFP staining only. **(O, O')** shows loss of Rab35 staining after knockdown of Rab35 in CCs. *UAS* activated at 30°C for 4 days (4d). Testes oriented with anterior at left. Image frames (A-R) 225μm and (N'-R') 112.5μm

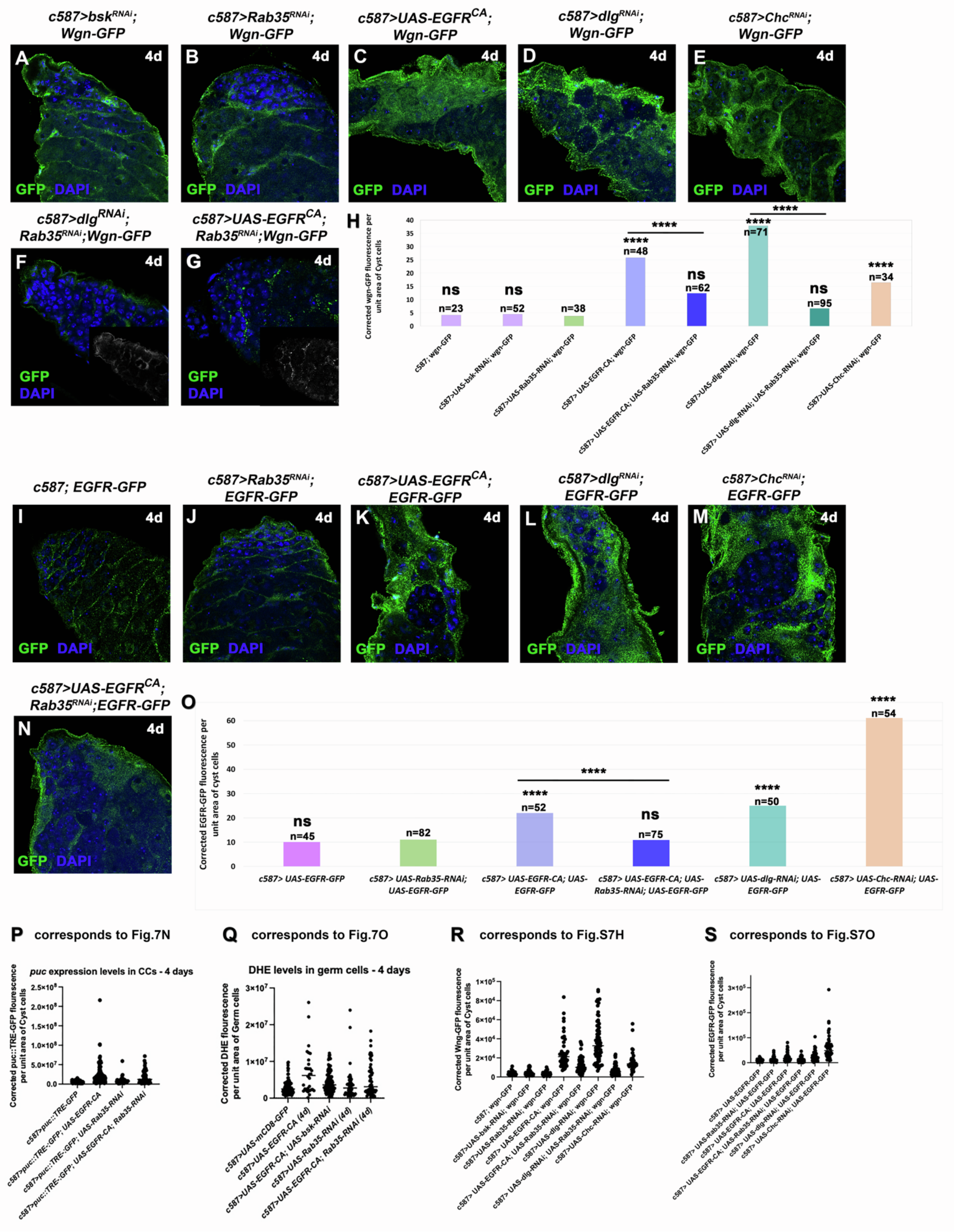

Figure S7: Rab35 is required in cyst cells to regulate Wgn and EGF receptor localization following EGFR overactivation, related to Figure 7**.** Adult testes of the indicated genotypes in the *Gal80*^{ts} background. **(A-G)** Wgn-GFP reflects JNK receptor Wengen localization in CCs. Wgn-GFP (green; CCs) and DAPI (blue; nuclei). Small inset picture in F and G show the Wgn-GFP staining

alone as levels are low to be easily seen in the merged pictures with DAPI. **(I-N)** *EGFR-GFP* reflects endogenous EGFR localization in CCs. EGFR-GFP (green; CCs) and DAPI (blue; nuclei). **(H, O)** Quantification of corrected fluorescent Wgn-GFP and EGFR-GFP levels in CCs, respectively. Each genotype was individually compared to control *c587>UAS-Rab35-RNAi,* while EGFR overactivation phenotypes (columns 4, 6 in S7H and column 3 in S7O) were compared to rescued ones (columns 5, 7 in S7H and column 4 in S7O), and showed significant reduction in the localization of both receptors upon rescue with *Rab35-RNAi* (error bars: standard error; ns: not significant; *$p<0.05$; **$p<0.01$; ***$p<0.001$; ****$p<0.0001$). Numbers (n) in each column represent sample size. **(P-S)** Dotted plots for quantifications of Figures 7N, 7O and the S7H, S7O (above). Testes oriented with anterior at left.
